# Comprehensive integration of diagnostic biomarker analysis and immune cell infiltration features in sepsis via machine learning and bioinformatics techniques

**DOI:** 10.3389/fimmu.2025.1526174

**Published:** 2025-03-10

**Authors:** Liuqing Yang, Rui Xuan, Dawei Xu, Aming Sang, Jing Zhang, Yanfang Zhang, Xujun Ye, Xinyi Li

**Affiliations:** ^1^ Department of Anesthesiology, Zhongnan Hospital of Wuhan University, Wuhan, Hubei, China; ^2^ Department of Anesthesiology, Hubei Provincial Engineering Research Center of Minimally Invasive Cardiovascular Sugery, Wuhan, China; ^3^ Department of Anesthesiology, Wuhan Clinical Research Center for Minimally Invasive Treatment of Structural Heart Disease, Wuhan, China; ^4^ Department of Geriatrics, Zhongnan Hospital of Wuhan University, Wuhan, Hubei, China

**Keywords:** sepsis, bioinformatics, machine learning, biomarkers, immune cell infiltration

## Abstract

**Introduction:**

Sepsis, a critical medical condition resulting from an irregular immune response to infection, leads to life-threatening organ dysfunction. Despite medical advancements, the critical need for research into dependable diagnostic markers and precise therapeutic targets.

**Methods:**

We screened out five gene expression datasets (GSE69063, GSE236713, GSE28750, GSE65682 and GSE137340) from the Gene Expression Omnibus. First, we merged the first two datasets. We then identified differentially expressed genes (DEGs), which were subjected to KEGG and GO enrichment analyses. Following this, we integrated the DEGs with the genes from key modules as determined by Weighted Gene Co-expression Network Analysis (WGCNA), identifying 262 overlapping genes. 12 core genes were subsequently selected using three machine-learning algorithms: random forest (RF), Least Absolute Shrinkage and Selection Operator (LASSO), and Support Vector Machine-Recursive Feature Elimination (SVW-RFE). The utilization of the receiver operating characteristic curve in conjunction with the nomogram model served to authenticate the discriminatory strength and efficacy of the key genes. CIBERSORT was utilized to evaluate the inflammatory and immunological condition of sepsis. Astragalus, Salvia, and Safflower are the primary elements of Xuebijing, commonly used in the clinical treatment of sepsis. Using the Traditional Chinese Medicine Systems Pharmacology Database and Analysis Platform (TCMSP), we identified the chemical constituents of these three herbs and their target genes.

**Results:**

We found that CD40LG is not only one of the 12 core genes we identified, but also a common target of the active components quercetin, luteolin, and apigenin in these herbs. We extracted the common chemical structure of these active ingredients -flavonoids. Through docking analysis, we further validated the interaction between flavonoids and CD40LG. Lastly, blood samples were collected from healthy individuals and sepsis patients, with and without the administration of Xuebijing, for the extraction of peripheral blood mononuclear cells (PBMCs). By qPCR and WB analysis. We observed significant differences in the expression of CD40LG across the three groups. In this study, we pinpointed candidate hub genes for sepsis and constructed a nomogram for its diagnosis.

**Discussion:**

This research not only provides potential diagnostic evidence for peripheral blood diagnosis of sepsis but also offers insights into the pathogenesis and disease progression of sepsis.

## Introduction

Sepsis, a life-threatening condition caused by a dysregulated host response to infection, remains a critical challenge in modern medicine (1). It is characterized by systemic inflammation and organ dysfunction, leading to high morbidity and mortality rates worldwide (2). Despite significant advancements in understanding its pathophysiology and improvements in clinical management, sepsis continues to impose a substantial burden on healthcare systems (3). Early diagnosis and effective treatment are often hindered by the heterogeneity of the condition and the lack of reliable biomarkers, underscoring the urgent need for innovative approaches to improve patient outcomes.

The complexity of sepsis lies in its multifaceted nature, involving intricate interactions between immune dysregulation, inflammatory cascades, and cellular dysfunction (4). Recent research has increasingly focused on identifying key molecular signatures and pathways that drive sepsis progression, with the aim of uncovering potential diagnostic markers and therapeutic targets (5). Advances in high-throughput sequencing technologies have revolutionized the study of sepsis by enabling comprehensive profiling of gene expression patterns, providing unprecedented insights into the molecular mechanisms underlying the disease (6). These technologies, combined with bioinformatics tools, have facilitated the analysis of large-scale datasets, revealing critical genes and pathways associated with sepsis pathogenesis (7).

The integration of machine learning algorithms has further enhanced the ability to analyze complex biological data, offering powerful tools for identifying robust biomarkers and predictive models (8). Techniques such as random forest, LASSO regression, and support vector machines have been employed to sift through vast amounts of genomic data, enabling the selection of key genes with diagnostic and prognostic potential (9). These approaches not only improve the accuracy of sepsis diagnosis but also pave the way for personalized treatment strategies by identifying patient-specific molecular profiles.

In parallel with these technological advancements, traditional Chinese medicine (TCM) has emerged as a promising complementary approach to sepsis management (10). Xuebijing, a traditional Chinese medicine injection widely used in clinical practice, is primarily composed of herbal ingredients such as safflower, salvia, and Astragalus (11). It is known for its effects in promoting blood circulation, removing blood stasis, clearing heat, and detoxifying (12). In recent years, Xuebijing has demonstrated significant efficacy in treating critical conditions such as sepsis, acute respiratory distress syndrome (ARDS), and multiple organ dysfunction syndrome (MODS) (13). Research indicates that Xuebijing exerts its therapeutic effects by inhibiting the release of inflammatory factors, improving microcirculation, and alleviating oxidative stress. For instance, a randomized controlled trial found that Xuebijing combined with conventional treatment significantly reduced the 28-day mortality rate in sepsis patients (14). Additionally, Xuebijing has shown potential in the treatment of COVID-19-related pneumonia, alleviating pulmonary inflammation and improving patient outcomes (15, 16). However, despite the positive results achieved in clinical applications, the specific mechanisms of action of Xuebijing require further in-depth research.

This study aims to bridge the gap between modern biomedical research and traditional medicine by exploring the molecular underpinnings of sepsis and the therapeutic potential of Xuebijing. By leveraging high-throughput sequencing, bioinformatics tools, and machine learning algorithms, we seek to identify key genes and pathways involved in sepsis pathogenesis. Furthermore, we aim to investigate the active components of Xuebijing and their molecular targets, integrating experimental validation to provide mechanistic insights. Our findings may not only advance the understanding of sepsis but also offer valuable insights into the development of personalized diagnostic and therapeutic approaches, ultimately improving outcomes for sepsis patients.

## Methods

### Gene expression datasets

The Gene Expression Omnibus (GEO) serves as a publicly accessible database for storing high-throughput gene expression data (https://www.ncbi.nlm.nih.gov/geo/), complete with tools for querying, downloading, and analyzing experiments as well as curated gene expression profiles. We scoured the GEO database using the keywords “Sepsis” [Mesh] AND “Expression profiling by array” [All Fields] AND “Homo sapiens” [porgn: txid9606]. Selection criteria included microarray datasets of whole-genome gene expression profiles from blood, with both sepsis and healthy samples. Each group had more than 12 samples. Ultimately, an in-depth examination was conducted on three distinct gene expression datasets. The specifics of these datasets are detailed in **Supplementary Table S1**. For the analysis, GSE69063 and GSE236713 were selected, while GSE28750, GSE65682 and GSE137340 served as the validation dataset.

### Detection of differentially expressed genes

Convert the probe IDs of the three datasets to gene symbols. Then, combine the two datasets GSE69063 and GSE236713 into one training set, and use the ‘removeBatchEffect’ function from the ‘limma’ package (17) (version 3.60.3) to eliminate batch effects between the datasets. Using the “limma” package to analyze combined data, we screened out differentially expressed genes (DEGs) in the sepsis group as compared to the control group, and plotted a volcano plot to delineate them. In GEO, the adjusted P values were examined to address the potential for false-positive results. |log2FC|> 1 and the adjusted P value < 0.05 were deemed to be the cutoffs for DEGs. We selected the top 25 genes with the highest and lowest logFC respectively among all statistically significant differentially expressed genes to create a heatmap utilizing the pheatmap package (version 1.0.12) in R software.

### Functional enrichment analysis

Gene Set Enrichment Analysis (GSEA) (18) serves as a computational method for assessing whether a specified group of genes exhibits a statistically notable, uniform disparity across two distinct biological conditions. This method pinpoints categories of genes or proteins that are excessively represented within an extensive collection of genes or proteins, potentially linking them to particular phenotypes and shedding light on intrinsic biological mechanisms. Kyoto Encyclopedia of Genes and Genomes (KEGG) is a comprehensive database that amalgamates genomic, biochemical, and phylogenetic information to systematically analyze gene functions and understand high-level biological functions and utilities. Gene Ontology (GO) analysis classifies genes into structured groups according to biological mechanisms, cellular elements, and molecular functions. After converting the IDs of the differentially expressed genes, we use the enrichGO and enrichKEGG functions from the clusterProfiler package (19) (version 4.12.0) for GO and KEGG analyses, respectively.

### Protein-protein interaction network

To explore further the molecular mechanisms underlying the onset and progression of sepsis, we selected differentially expressed genes with |log2FC| > 2. Using the STRING database (https://cn.string-db.org/), we analyzed the protein-protein interaction network to reveal the functions and mechanisms of proteins within cells, as well as the complex regulatory networks in biological systems. The PPI network was constructed based on empirically validated interactions, each having a cumulative score greater than 0.4.

### The weighted gene co-expression network

Weighted Gene Co-expression Network Analysis (WGCNA) (20) serves as a systemic biology technique for uncovering correlation patterns between different genes in microarray or RNA-Seq datasets. This method discerns groups (modules) of genes that are closely correlated and connects these modules to distinct external characteristics. The main steps include data input and preprocessing, network construction, module detection, and relating modules to external traits. To link modules with clinical features, we calculated the Module Membership (MM) and Gene Significance (GS) values. MM values exceeding 0.8 and GS values over 0.2 signify strong connectivity within modules and significant clinical association. Extract all strongly associated genes from each module for subsequent analysis.

### Identification and confirmation of diagnostic markers

We utilized three machine learning methods, including Random Forest (RF), Least Absolute Shrinkage and Selection Operator (LASSO) logistic regression, and Support Vector Machine-Recursive Feature Elimination (SVM-RFE), to identify key biomarkers linked with sepsis. Random forest is an ensemble learning method that improves the generalization ability of the model by constructing 500 decision trees and performing voting or averaging. The seed is set to 19991018 to ensure reproducibility. The importance score of each feature is calculated based on the Gini index. For the random forest analysis, we used the “randomForest” package (version 4.7.1.1) in R.Lasso regression is a linear regression method used for feature selection and regularization. It introduces an L1 regularization term to penalize the complexity of the model, causing the coefficients of some unimportant features to become zero. The model is specified as a binary classification model with L1 regularization. It calculates 100 different λ values along the regularization path. Mean squared error (MSE) is used as the evaluation metric for cross-validation. Five-fold cross-validation is employed to select the optimal λ value. Genes with non-zero coefficients are selected. For the LASSO logistic regression analysis, we used the “glmnet” package (version 4.1.8) in R.SVM-RFE is a feature selection method based on support vector machines (SVM) that recursively eliminates the least important features to select the optimal feature subset. Five-fold cross-validation is used. When the number of features exceeds 100, half of the features are eliminated in each iteration. SVM-RFE achieves feature selection by selecting the feature subset with the lowest error rate. For the SVM-RFE analysis, we used the “e1071” package (21) (version 1.7.14) in R.

### Molecular docking

The molecular structures of active ingredients were sourced from the PubChem database and subsequently imported into ChemBio3D 14.0 for spatial conformation adjustment of the active ingredients, energy optimization computations, and ultimately saved in the mol2 file format. Following processing with AutoDockTools 1.5.6, these files were converted and saved in pdbq format. The three-dimensional crystal structure of the target protein was obtained from the Uniprot database. Using Notepad2, the water molecules and organic substances were excised from the target protein. Subsequently, the protein was imported into AutoDockTools (version 1.5.6) to undergo hydrogenation, charge assignment, and atomic type assignment, culminating in the saving of a pdbqt format file. Molecular docking was executed with AutoDockVina, and the resultant docking images were generated using Pymol 2.6.

### Clinical study on patients with sepsis

Between September 2024 and December 2024, a total of 12 patients with sepsis (age >18 years) were enrolled from the Department of Intensive Care Unit at Zhongnan Hospital of Wuhan University. The inclusion criteria for sepsis were based on the Third International Consensus Definitions for Sepsis and Septic Shock (Sepsis-3): suspected or confirmed infection, an acute increase of ≥ 2 points in the Sequential Organ Failure Assessment (SOFA) score, and evidence of organ dysfunction or tissue hypoperfusion. Being younger than 18 years of age, pregnant, or nursing, and having malignant tumors were all excluded from participation. Collect whole blood samples from 6 non-infected patients undergoing surgery in the Department of Anesthesiology at Zhongnan Hospital of Wuhan. We obtained the permission from the Ethics Committee of Zhongnan Hospital of Wuhan University, and participants gave their informed consent at the start of the research (the ethics batch number: 2024260k).

### Isolation of PBMCs from whole blood

Peripheral blood mononuclear cells (PBMCs) were isolated from whole blood samples using density gradient centrifugation. Following the protocol described in previous literature, human peripheral blood was collected into anticoagulant tubes and gently inverted to prevent clotting. The anticoagulated whole blood was diluted 1:1 with PBS and mixed gently.

A 15 mL sterile centrifuge tube was filled with 4 mL of lymphocyte separation medium (Ficoll-Paque PLUS). The diluted whole blood was carefully layered on top of the separation medium, ensuring that the interface between the two layers remained intact and undisturbed. The tube was then placed in a horizontal rotor and centrifuged at 400 × g for 30 minutes at room temperature (with no brake). After centrifugation, the intermediate white layer (PBMCs) was carefully aspirated using a sterile pipette and transferred to a new 15 mL centrifuge tube. Ten milliliters of PBS were added, and the mixture was gently resuspended and centrifuged at 300 × g for 10 minutes at room temperature. The supernatant was discarded, and the PBMCs were resuspended in DMEM growth medium (Gibco, 11965118) containing 2% fetal bovine serum or in Cryostor CS10 (StemCell Technologies) for overnight storage at -80°C, followed by long-term storage in liquid nitrogen.

### qRT-PCR analysis

Total RNA was derived from human PBMC using TRIzol reagent (Invitrogen, USA). The RNA was subsequently converted into cDNA through reverse transcription, utilizing the reverse transcription kit (produced by Takara, China). cDNA was used as a template for qPCR, with target gene primers and reference gene primers added. The reaction mixture was prepared adhered to the guidelines provided by the qPCR kit (Takara, China). Ultimately, the expression levels of the target gene were determined by analyzing the qPCR outcomes with the Bio-Rad CFX Maestro software. The sequences of the primers can be found in “**Supplementary Table S2**”.

### Western blot

Human PBMCs were cultivated in DMEM medium supplemented with 10% fetal bovine serum and 1% penicillin-streptomycin, within a regulated incubator atmosphere maintained at 37°C and 5% CO2. Protein extraction was performed using RIPA buffer, which included inhibitors for proteases and phosphatases, followed by protein quantification via the BCA method. Identical amounts of proteins were separated on SDS-PAGE gels and subsequently blotted onto PVDF membranes. The membranes were blocked with 5% non-fat milk in TBST, before being exposed to the anti-CD40LG antibody (abcam, ab2391) overnight at 4°C. Afterward, HRP-conjugated secondary antibodies were applied. Protein bands were detected by ECL and visualized with a chemiluminescence imager. ImageJ software was utilized to measure the intensity of the bands.

### Cecal ligation and puncture induced sepsis model in mice

Male C57BL/6 mice (8 weeks old) were used to establish the sepsis model via cecal ligation and puncture (CLP). After anesthesia with 1.0% pentobarbital sodium injections intraperitoneally (35 mg/kg), a midline incision was made to expose the cecum. The cecum was ligated below the ileocecal valve and punctured twice with a 21-gauge needle. A small amount of fecal matter was extruded to ensure patency, and the cecum was then returned to the abdominal cavity. The incision was closed in two layers, and the mice were resuscitated with 1 ml of pre-warmed saline subcutaneously. Sham-operated mice underwent the same procedure without cecal ligation and puncture. Post-surgery, all mice were monitored closely for signs of sepsis. Flavonoids (5 mg/kg, obtained from Song Hui Laboratory) were administered via tail vein injection to evaluate their protective effects on sepsis-induced organ damage. Every experiment was carried out in accordance with NIH guidelines and approved by Wuhan University’s Animal Ethics Committee (ZN2021185).

### Liver function-related index detection

Whole blood samples were left at room temperature for 2 hours and then centrifuged at 3000 rpm for 15 minutes at 4°C to obtain serum. Liver function tests were performed using an enzymatic colorimetric method, including alanine aminotransferase (ALT) (BioBASE 70111), aspartate aminotransferase (AST) (BioBASE 70910). Enzymatic colorimetric assay reagents were prepared according to the kit instructions. Samples and reagents were incubated at 37°C. The samples were loaded onto an automatic biochemical analyzer (Shandong Brocade Biotechnology Co., Ltd., BK-280) for automatic measurement. Absorbance values of each indicator were measured using a spectrophotometer. The concentrations of each liver function indicator were calculated based on the standard curve.

### Concentration in bronchoalveolar lavage fluid

According to previous manufacturer’s protocol (22), lungs of mice were lavaged after experiment. Using a commercial bicinchoninic acid (BCA) protein assay kit (Beyotime, China), the proteins of BALF were quantified.

### Assessment and correlation study of immune cells related to infiltration

The CIBERSORT website approximates the copiousness of 22 distinct immune cell varieties within composite cell populations by analyzing gene expression data. The immune cell infiltration matrix was obtained, as demonstrated by a p-value less than 0.05. Use the “ggplot2” (version 3.5.1) and “ggcorrplot” (version 0.1.4.1) packages to plot the heatmap of immune cell distribution and the correlation matrix of immune cell distribution, respectively. Finally, we conducted a correlation between pivotal genes and immune cells that have infiltrated the tissue.

### Software tools

The analysis in this study was based on R version 4.4.0 and utilized the following key R packages and their versions: ggplot2 (version 3.5.1) for data visualization, limma (version 3.60.3) (17) for differential expression analysis, and WGCNA (version 1.72-5) (20) for weighted gene co-expression network analysis.

### Statistical methods

All statistical tests were performed using GRAPHPAD 6.0c. Results are expressed as mean ± SD. Comparisons between the experimental and control groups were made using independent sample t-tests or one-way analysis of variance (ANOVA). A p-value of less than 0.05 was considered statistically significant.

## Results

### Screening of differentially expressed genes in sepsis

**Figure 1:**
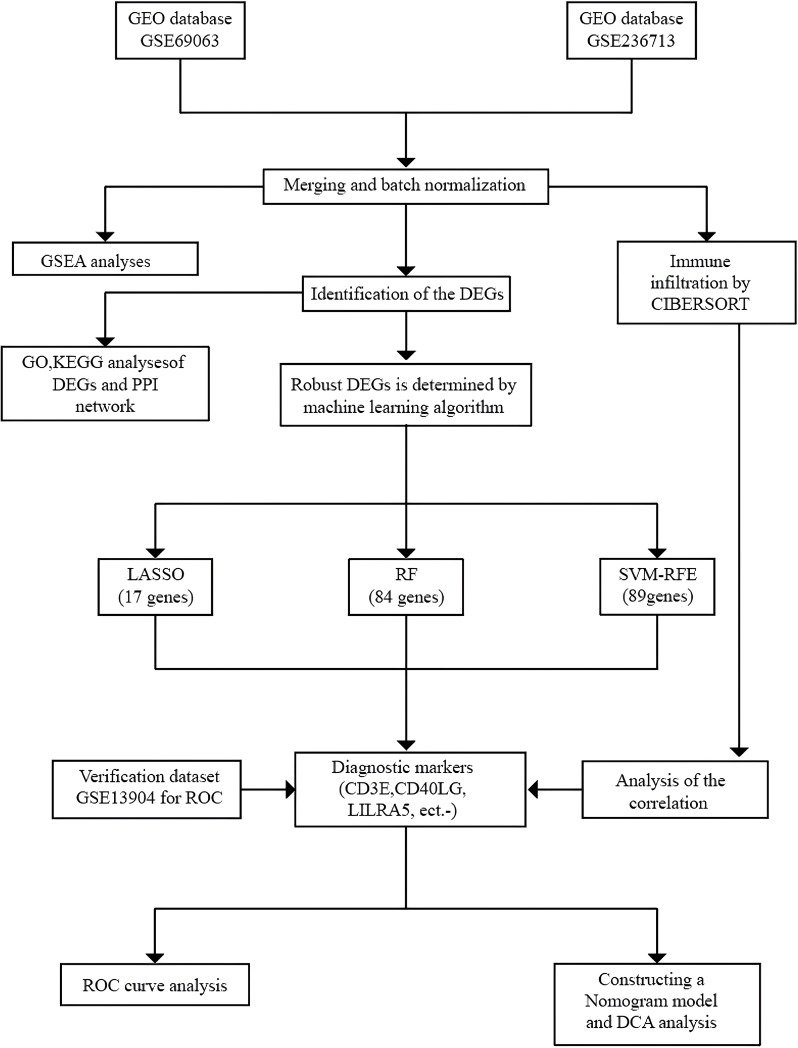
The flowchart portraying the investigation procedure. GEO, Gene Expression Omnibus; GSEA, Gene Set Enrichment Analysis; CIBERSORT, Cell-Type Identification by Estimating Relative Subsets of RNA Transcripts; DEGs, Differentially Expressed Genes; GO, Gene Ontology; KEGG, Kyoto Encyclopedia of Genes and Genomes; PPI, Protein-Protein Interaction; LASSO, Least Absolute Shrinkage and Selection Operator; RF, Random Forest; SVM-RFE, Support Vector Machine-Recursive Feature Elimination; ROC, Receiver Operating Characteristic Curve; DCA, Decision Curve Analysis.

The procedure of this research is depicted in **Figure 1**, and the relevant information is provided in **Supplementary Table S1**. We combined the two datasets GSE69063 and GSE236713 into one training set, and use the ‘removeBatchEffect’ function from the ‘limma’ package to eliminate batch effects between the datasets (**Supplementary Figure S1**). We employed the “limma” package (17) (version 3.60.3) to identify differentially expressed genes (DEGs) with |log_2_FC| > 1 and an adjusted p-value < 0.05 as cutoffs, resulting in a total of 1443 genes, including 891 upregulated and 552 downregulated genes. We selected the top 25 genes with the highest and lowest logFC respectively among all statistically significant differentially expressed genes to create a heatmap (**Supplementary Figure S2A**) by using R software’s pheatmap package. A graphical representation, known as a volcano plot, was created to highlight the varying expression levels of the differentially expressed genes (DEGs). (**Supplementary Figure S2B**).

### GSEA

**Figure 2:**
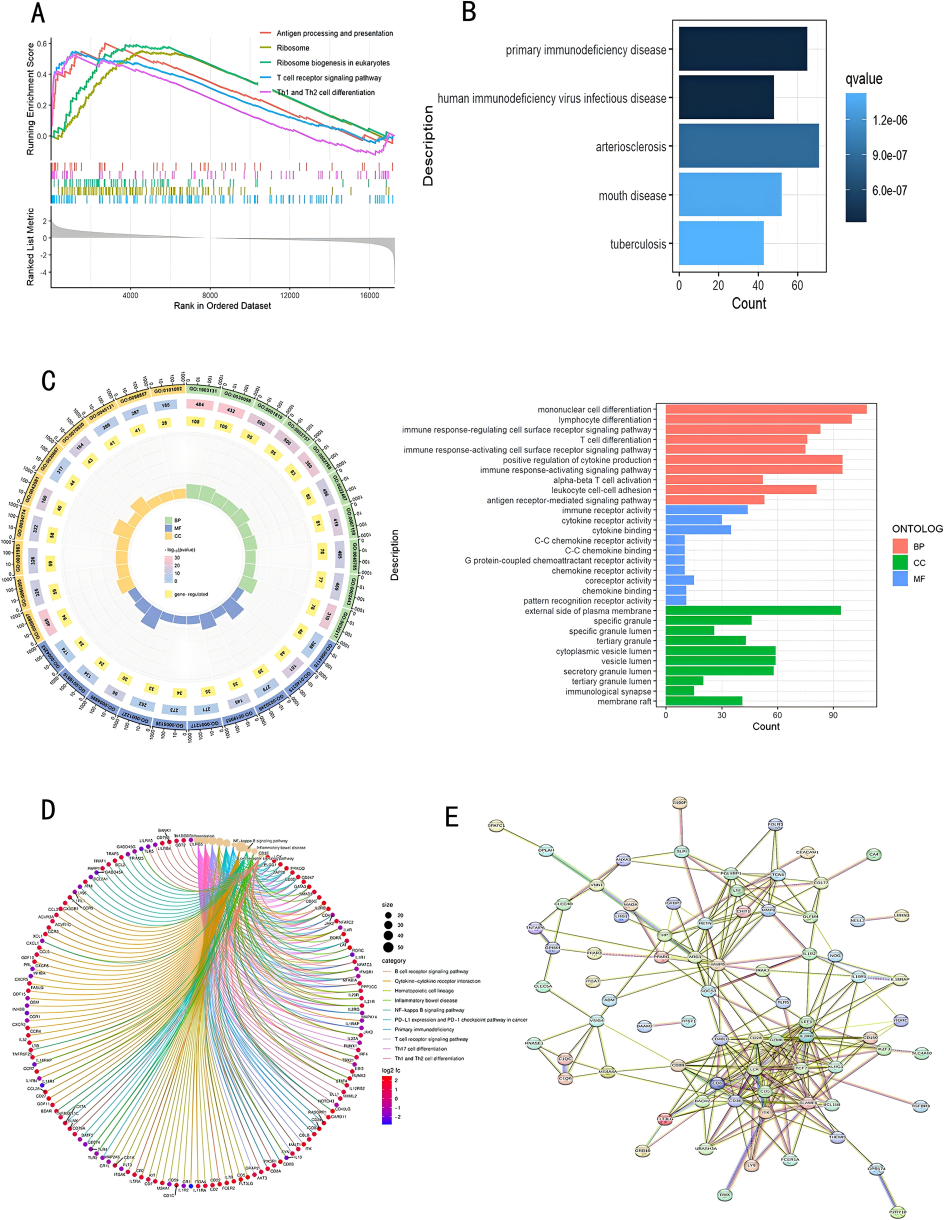
Functional and pathway enrichment assessment of DEGs. **(A)** GSEA evaluation; **(B)** DO examination; **(C)** GO enrichment evaluation; **(D)** KEGG pathway enrichment evaluation; **(E)** PPI network analysis. DEGs, Differentially Expressed Genes; GSEA, Gene Set Enrichment Analysis; DO, Disease Ontology; GO, Gene Ontology; KEGG, Kyoto Encyclopedia of Genes and Genomes; PPI, Protein-Protein Interaction.

To study the relationship between genes and specific phenotypes in sepsis patients and healthy controls using GSEA, we identified potential biological processes. Through HALLMARK analysis, we found that the top-ranked terms were mostly related to antigen presentation, cell differentiation, immune response, and other processes. In sepsis patients, the signaling pathways for antigen processing and presentation, ribosome, ribosome biogenesis in eukaryotes, T cell receptor signaling pathway, as well as the differentiation of Th1 and Th2 cells, with all the adjusted p-values falling below 0.05. (**Figure 2A**)

### Functional enrichment analysis of DEGs

Based on the findings from functional enrichment analysis, DEGs pertained to conditions including primary immunodeficiency disease, HIV infection, arteriosclerosis, oral diseases, and tuberculosis (**Figure 2B**). According to the output of GO enrichment analysis, DEGs are mainly associated with T cell differentiation, immune response activation of cell surface receptors, innate immune response activation signal transduction, immune receptor activity, and extracellular matrix-related functions. **Figure 2C** shows the enrichment results for these functions. The KEGG analysis is related to Th17 cell differentiation, Th1 and Th2 cell differentiation, T cell receptor signaling pathway, and Cytokine-cytokine receptor interaction (**Figure 2D**). **Figure 2E** shows the PPI network, in which core proteins such as CD5, CD3E, CD6, LCK, etc., play crucial roles in T cell activation and signal transduction.

### Detection of co−expression gene modules within sepsis

**Figure 3:**
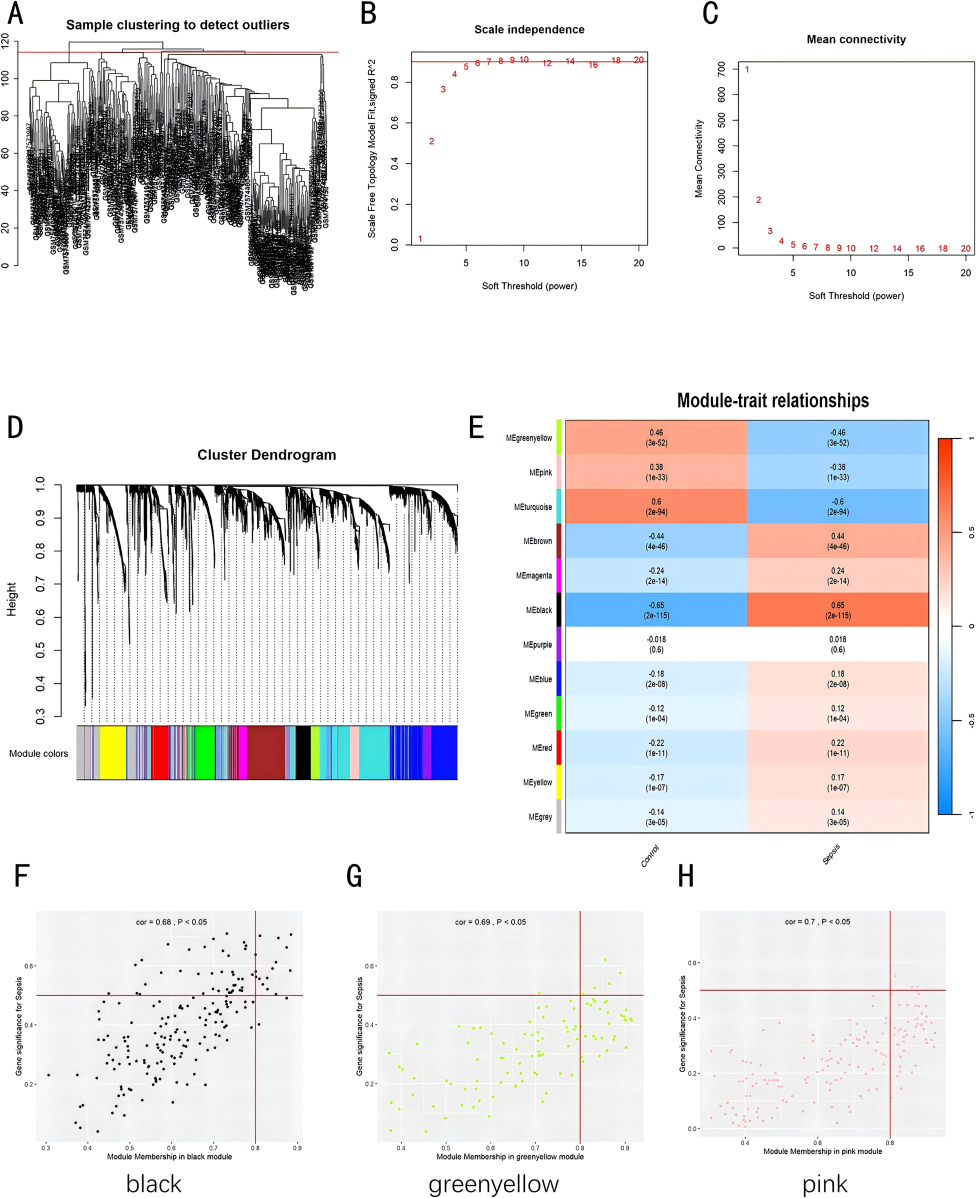
Construction of weighted co-expression network datasets in pediatric sepsis. **(A)** Clustering dendrogram of 226 samples; **(B, C)** Evaluation of network topology for different soft thresholds (β); **(D)** Gene dendrograms derived from average linkage hierarchical clustering; **(E)** Module-trait correlations; **(F)** Scatterplot of GS for recurrence vs. MM in the black module; **(G)** Scatterplot of GS for recurrence vs. MM in the greenyellow module; **(H)** Scatterplot of GS for recurrence vs. MM in the pink module. GS, Gene Significance; MM, Module Membership.

In the training dataset, we applied WGCNA to identify gene modules extensively co-expressed across numerous genes. First, the samples from two datasets were divided into two groups: sepsis group and control group. A cutHeight of 114 was set to remove outliers (**Figure 3A**). Then, the minimum soft threshold with a scale-free topology model fit close to 0.9 was chosen, with 5 as the soft threshold power (β), for constructing biologically significant scale-free networks in the next step (**Figures 3B, C**). Through employing hierarchical clustering and dynamic branch cutting techniques to dissect gene dendrograms, we divided genes into 12 modules (**Figure 3D**). The modules of black, greenyellow, and pink exhibited a significant correlation with sepsis (**Figure 3E**, P < 0.05). We plotted scatter plots for all modules with p-values indicating significant differences (p < 0.05) (**Supplementary Figure S3**). **Figures 3F–H** show scatter plots for the three most significantly correlated modules. We extracted genes from all modules with threshold of membership > 0.8 and threshold of significance > 0.2. A total of 351 genes showed significant correlations with sepsis-related genes and module membership.

### Assessment and verification of diagnostic biomarkers

**Figure 4:**
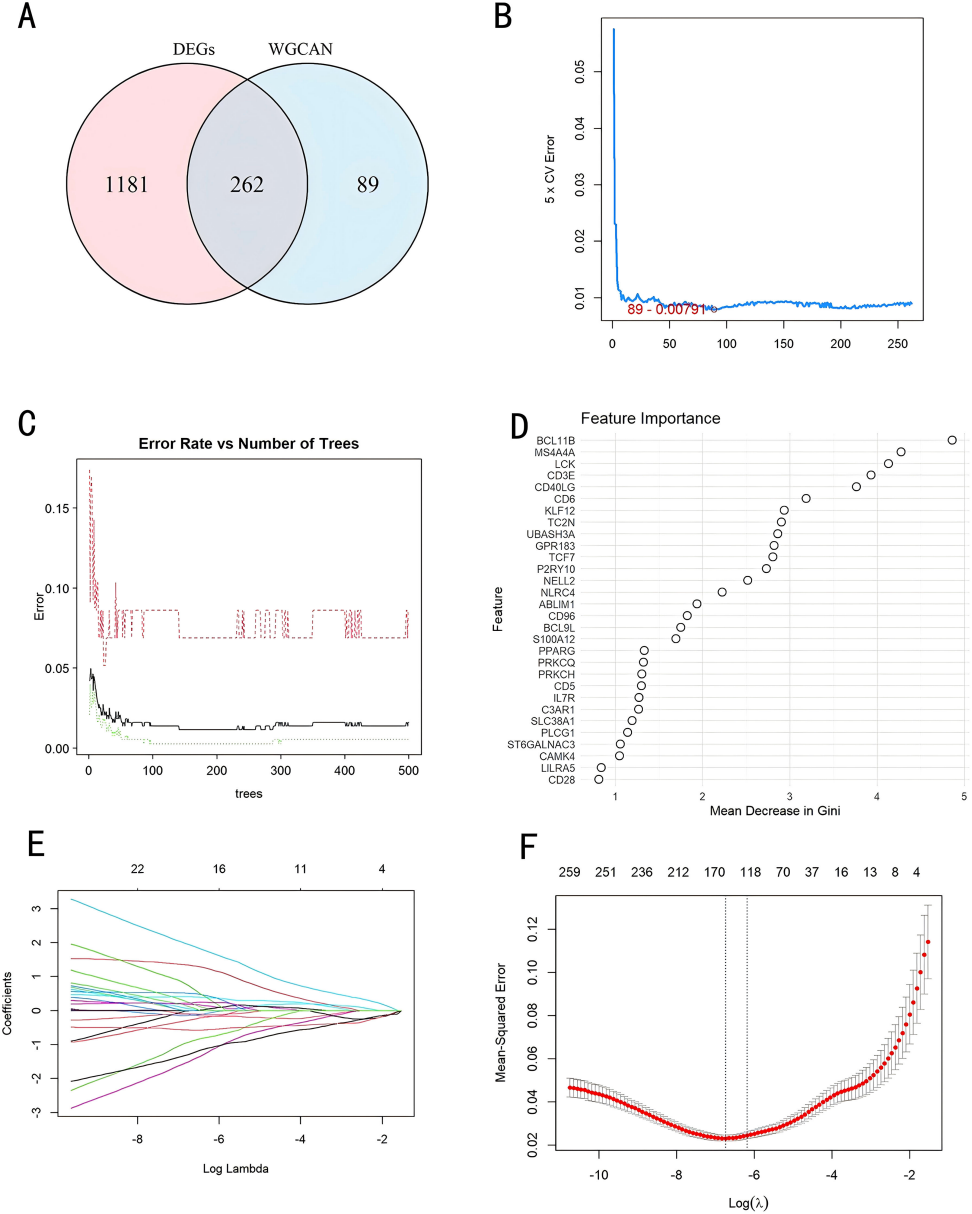
Identification of diagnostic markers through a comprehensive approach. **(A)** Venn diagram comparing key module genes with DEGs; **(B)** Biomarker screening via SVM-RFE; **(C, D)** Based on RF algorithm to screen biomarkers; **(E)** Varied colors indicate distinct genes; **(F)** Diagnostic marker screening employing the LASSO logistic regression algorithm. DEGs, Differentially Expressed Genes; WGCNA, Weighted Gene Co-Expression Network; SVM-RFE, Support Vector Machine-Recursive Feature Elimination; RF, Random Forest; LASSO, Least Absolute Shrinkage and Selection Operator.

**Figure 5:**
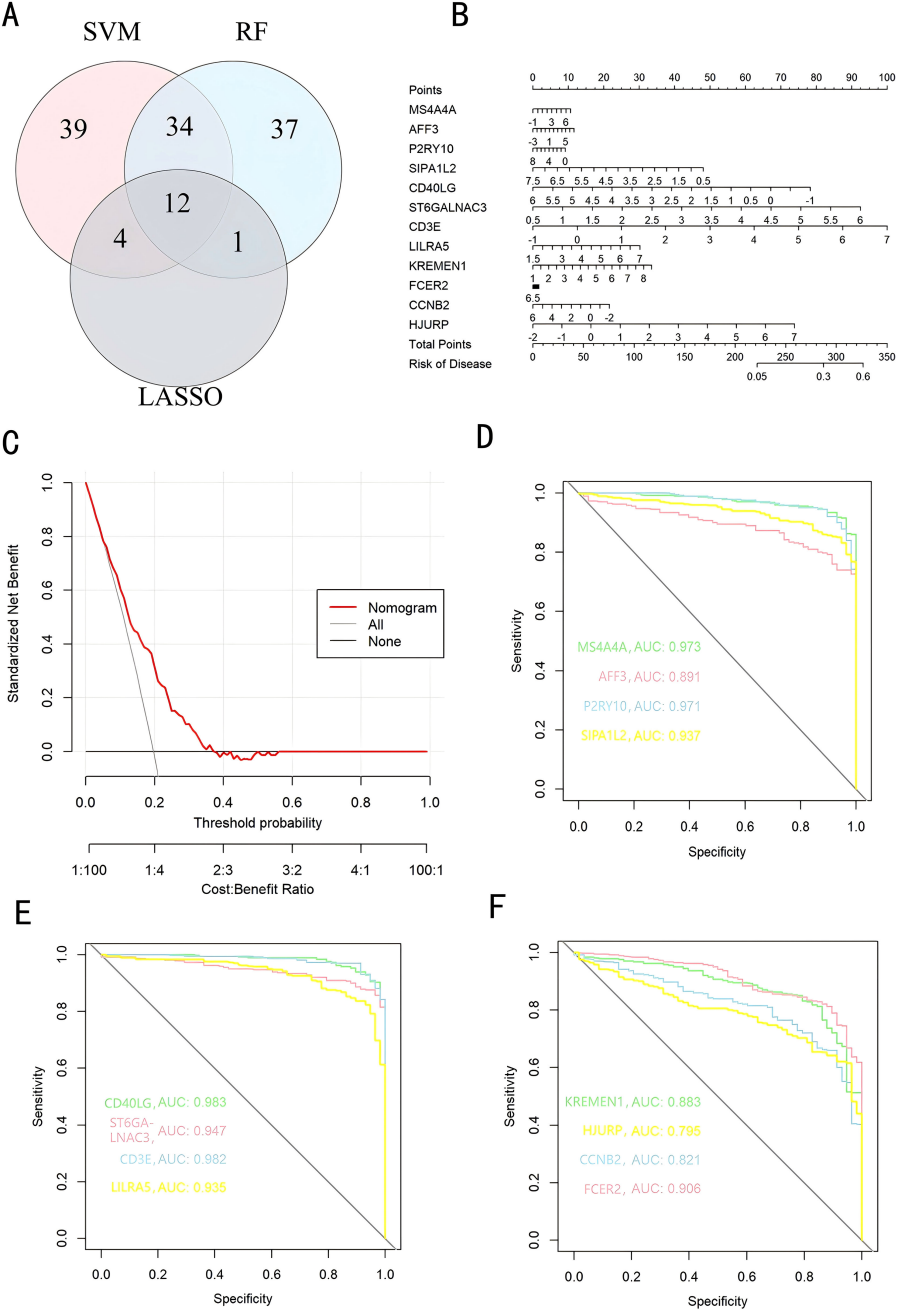
Key Genes in Diagnosing Pediatric Sepsis. **(A)** A Venn diagram illustrates the overlap of diagnostic markers identified by the three algorithms; **(B)** A nomogram is employed to forecast the incidence of pediatric sepsis; **(C)** Decision Curve Analysis (DCA) plots; **(D-F)** The ROC curve for validating diagnostic effectiveness. DCA, Decision Curve Analysis; ROC, Receiver Operating Characteristic Curve; AUC, Area Under Curve.

**Figure 6:**
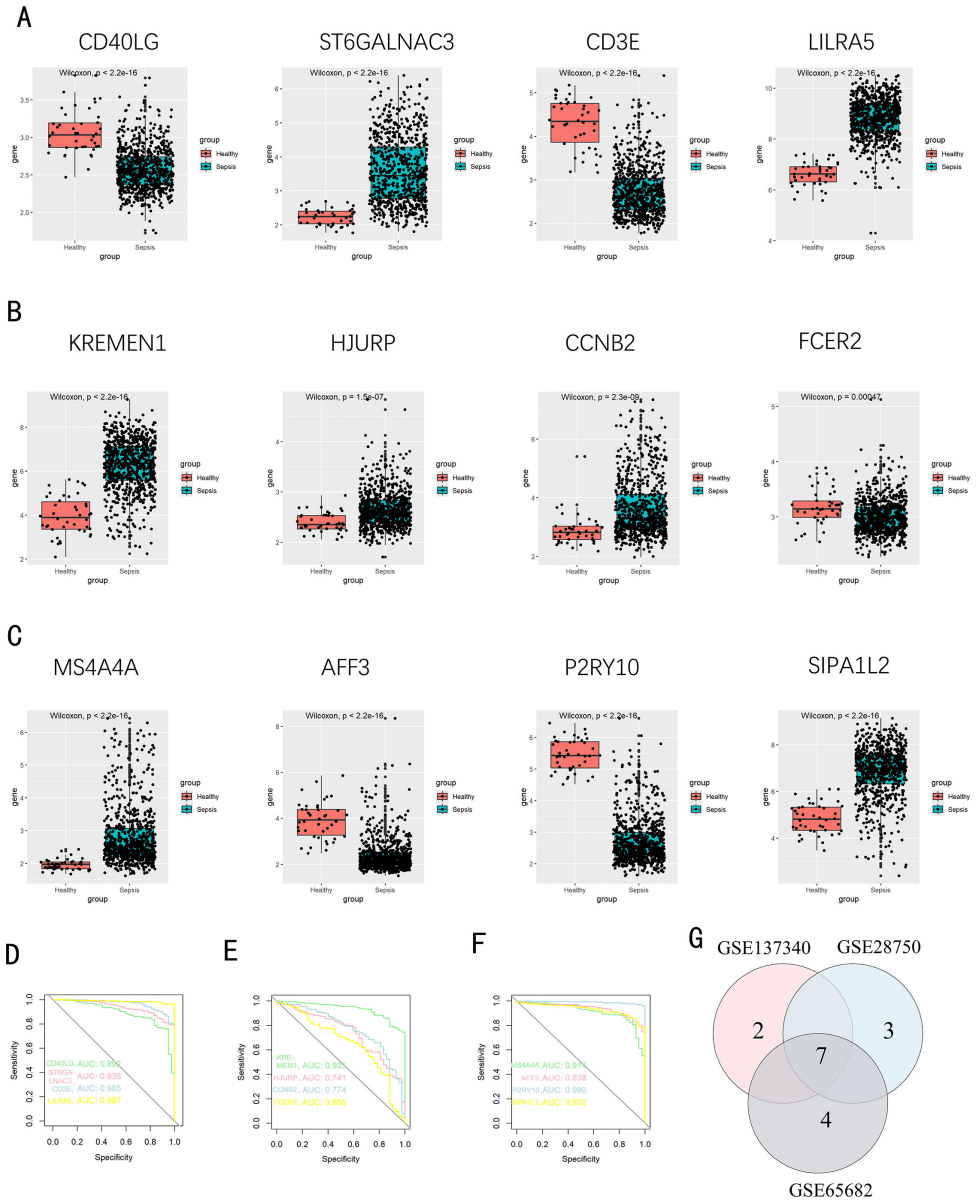
Confirmation of pivotal genes. **(A-C)** A boxplot depicts the expression levels of key genes between the pediatric sepsis group and the control group in GSE65682 dataset; **(D-F)** The ROC curve for diagnostic efficacy validation in GSE65682. **(G)** A Venn diagram illustrates the overlapping genes with significant differences and AUC greater than 0.7 and single-gene boxplot P-values less than 0.05 in all validation sets.

We used a Venn diagram to display the overlapping genes between DEGs and key modules identified by WGCNA, resulting in a total of 262 shared genes (**Figure 4A**). Three machine learning algorithms were applied to pinpoint characteristic genes: the SVM-RFE error rate graph indicates that when 89 genes are selected, the diagnostic error rate is the lowest (**Figure 4B**); **Figure 4C** shows the fitting process of the Random Forest algorithm. The RF accuracy graph indicates that when the number of feature genes is 84, the diagnostic accuracy is the highest (**Supplementary Figure S2C**). In the Random Forest model, the top 30 feature genes are ranked in descending order of importance (**Figure 4D**); LASSO regression was applied to identify 17 genes predictive of the outcome, following univariate statistical significance assessments. (**Figures 4E, F**). We extracted 12 feature genes (MS4A4A, AFF3, P2RY10, SIPA1L2, CD40LG, ST6GALNAC3, CD3E, LILRA5, KREMEN1, FCER2, CCNB2, HJURP) covered by all three machine learning algorithms, and illustrated their overlap with a Venn diagram (**Figure 5A**). Utilizing the “rms” package (version 6.8.1), we crafted nomogram models for the diagnosis of sepsis, leveraging the data from these 12 genes. (**Figure 5B**) Based on the decision curve analysis (DCA) outcomes, the Nomogram model yields superior clinical advantages (**Figure 5C**). In the training dataset, the AUC for all 12 feature genes exceeds 0.7, indicating their high predictive accuracy and suggesting their potential as clinical biomarkers (**Figures 5D–F**). In the GSE65682 validation group, all 12 core genes showed significant differences between the healthy control group and the sepsis group (**Figures 6A–C**). Except for FCRE2, the area under the ROC curve (AUC) for the remaining core genes was above 0.7(**Figures 6D–F**). In the three validation datasets, we selected genes with AUC greater than 0.7 and single-gene boxplot P-values less than 0.05 in each dataset to create a Venn diagram. We found that there were 7 overlapping genes, among which CD40LG was one (**Figure 6G**). Moreover, in the GSE137340 validation group, the levels of CD3E, CD40LG, FCER2, and P2RY10 expressions were markedly reduced in the sepsis group when contrasted with the control group (P < 0.05) (**Supplementary Figure S5A**). Conversely, the expression of CCNB2, HJURP, KREMEN1, LILRA5, and SIPA1L2 was significantly higher in the sepsis group (P < 0.05) (**Supplementary Figure S5B**). The remaining genes exhibited no substantial disparity when comparing the two groups. In the validation dataset, ROC curves were plotted for 12 genes (**Supplementary Figures S5C–E**). Except for MS4A4A, AFF3, and ST6GALNAC3, the AUC of the remaining genes’ curves was greater than 0.7. We used a Venn diagram to show overlapping genes (CD3E, CD40LG, LILRA5 and FCER2) with significant differences and an ROC curve area greater than 0.8 in the validation dataset (**Supplementary Figure S5F**). Similarly, in the GSE28750 dataset, we performed single-gene boxplot validation (**Supplementary Figures S6AC–C**) and ROC curve validation (**Supplementary Figures S6DC–F**) for the 12 core genes.

### Infiltration of immune cells results

**Figure 7:**
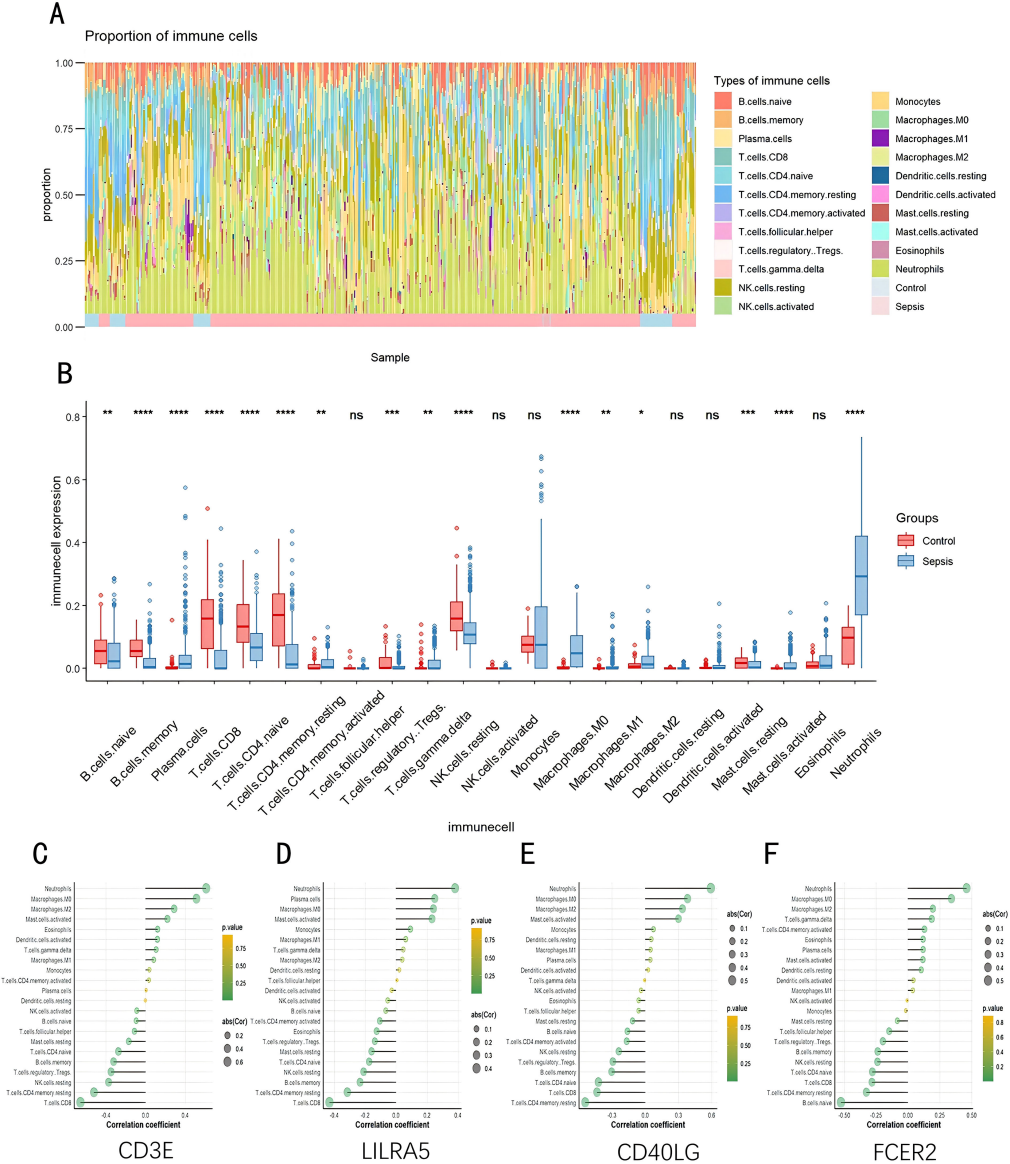
Evaluation, visualization, and correlation analysis of immune cell infiltration. **(A)** Boxplot and **(B)** Violin plot depicting the distribution of 22 distinct immune cell types. **(C)** Linkage between CD3E and infiltrating immune cells; **(D)** Linkage between LILRA5 and infiltrating immune cells; **(E)** Linkage between CD40LG and infiltrating immune cells; **(F)** Linkage between FCER2 and infiltrating immune cells. NK, natural killer. *p < 0.05, **p < 0.01, ***p < 0.001, ****p < 0.0001, ns stands for non-significant.

As indicated by the CIBERSORT analysis, septic specimens generally exhibit an elevated prevalence of plasma cells, CD4+ T cells in the memory activated state, gamma delta T cells, M0 Macrophages, M2 Macrophages, and Neutrophils, as compared with normal controls (P < 0.05) (**Figures 7A, B**). However, B cells naive, B cells memory, T cells CD8, T cells CD4 naive, T cells CD4 memory resting, and NK cells resting are relatively lower in septic samples. Correlation analysis results indicate significant associations of CD3E, CD40LG, LILRA5 and FCER2 with various immune cells (**Figures 7C–F**). The Correlation analysis results of the remaining genes are shown in **Supplementary Figure S4**.

### Screening of therapeutic targets

**Figure 8:**
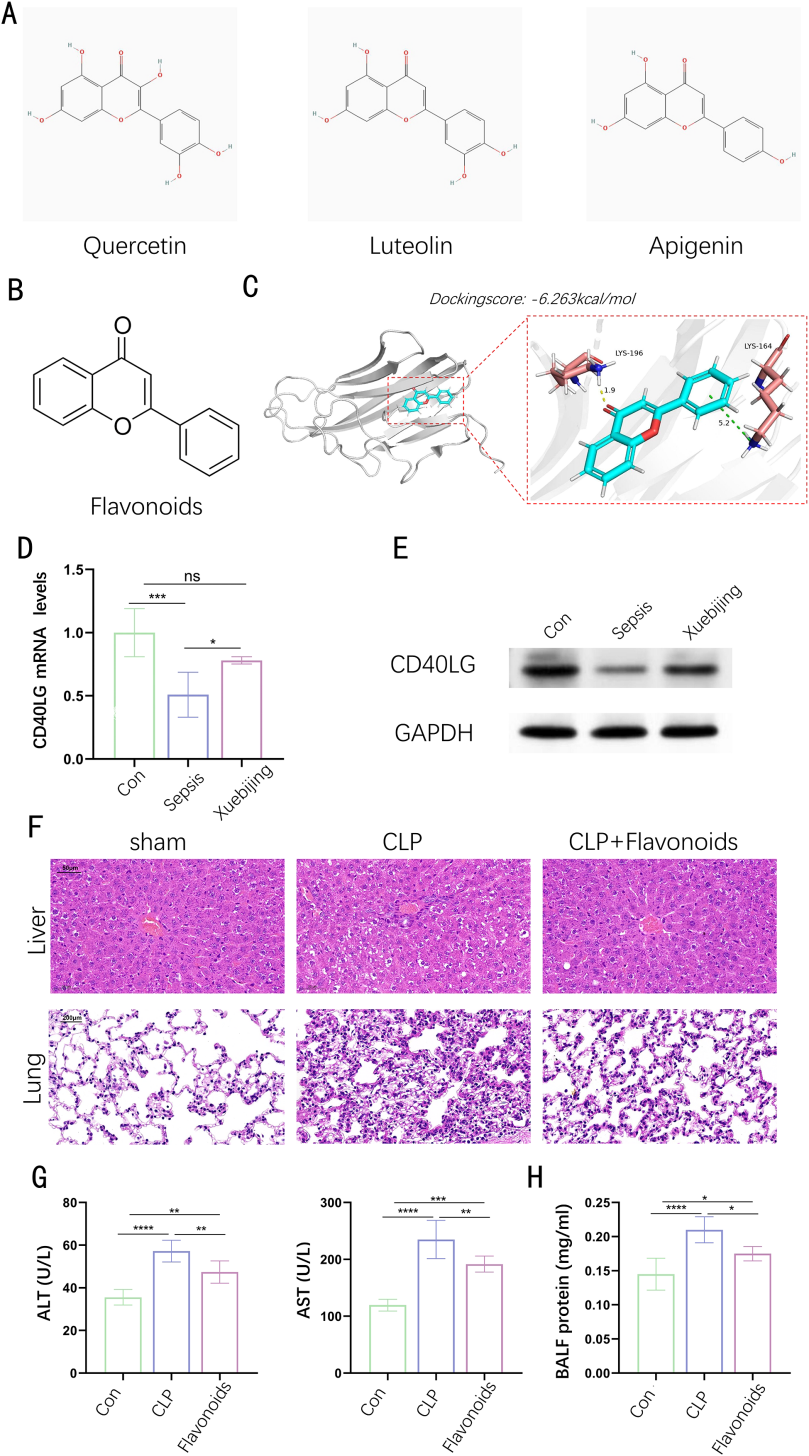
Interaction between Flavonoids and CD40LG. **(A)** The chemical structures of quercetin. Luteolin and apigenin. **(B)** The chemical structure of flavonoids. **(C)** 3D Binding model analysis of CD40LG with flavonoids. **(D)** The relative expression of CD40LG mRNA in control (Con), sepsis, and Xuebijing-treated groups. **(E)** Western blot analysis of CD40LG and GAPDH protein expression in control (Con), sepsis, and Xuebijing-treated groups. **(F)** Liver tissue sections stained with hematoxylin and eosin (H&E), showing the liver and lung tissue structure of the sham operation group, cecal ligation and puncture (CLP) group, and CLP plus flavonoid treatment group. **(G)** Serum ALT and AST levels in different treatment groups. **(H)** Protein concentration in bronchoalveolar lavage fluid (BALF) in different treatment groups. (n=6). Data are presented as mean ± SD. Statistical significance is indicated as follows: ns (not significant), *p < 0.05, **p < 0.01, ***p < 0.001, ****p < 0.0001, ns stands for non-significant.

Xuebijing, a traditional Chinese medicine injection composed of safflower, salvia, and Astragalus, is widely used for its ability to promote blood circulation, clear heat, and detoxify. It has shown significant efficacy in treating critical conditions such as sepsis, ARDS, and MODS, primarily by inhibiting inflammatory factors, improving microcirculation, and reducing oxidative stress. While clinical studies have demonstrated its potential to reduce mortality and alleviate conditions like COVID-19-related pneumonia, the precise mechanisms of Xuebijing’s action remain to be fully elucidated. Employing the Traditional Chinese Medicine Systems Pharmacology Database and Analysis Platform (TCMSP), we discerned the chemical components found in the three herbs along with their corresponding target genes. We found that CD40LG is not only one of the 12 core genes we identified, but also a common target of the active components quercetin, luteolin, and apigenin in these herbs. **Figure 8A** illustrates the chemical structures of these active ingredients, and we extracted the common chemical structure of them—flavonoids (**Figure 8B**). The 3D binding model analysis of CD40LG with flavonoids shows a docking score of -6.263 kcal/mol, indicating strong affinity between the compound and the protein, as values more negative than -5 reflect good affinity (**Figure 8C**). Through docking analysis, we further validated the interaction between flavonoids and CD40LG, providing strong evidence for personalized sepsis treatment. To further substantiate the clinical applicability of the gene in practical scenarios and explore its roles in the pathogenesis of sepsis, we collected blood samples from both a healthy control group and a sepsis group with or without the administration of Xuebijing. We extracted PBMCs from blood samples and conducted WB and PCR experiments on them. At both gene and protein levels, CD40LG significantly decreased in sepsis patients without Xuebijing treatment compared to the healthy group, while CD40LG was partially rescued in sepsis patients with Xuebijing treatment (**Figures 8D, E**). Using the sham operation group as a control, we established a mouse sepsis model through cecal ligation and puncture (CLP) and administered flavonoids to a portion of the septic mice via tail vein injection. We observed the histological changes in the liver and lung tissues among different treatment groups. The results showed that CLP treatment exacerbated liver and lung injuries in mice, while flavonoid treatment effectively alleviated the histological changes in the liver and lung tissues (**Figure 8F**). Similarly, the changes in serum ALT and AST levels (**Figure 8G**) and BALF protein concentration (**Figure 8H**) among the three groups followed the same trend: CLP treatment significantly increased ALT, AST, and BALF protein concentrations, whereas flavonoid treatment significantly decreased these indices. These results indicate that flavonoids have a protective effect against CLP-induced tissue injury.

## Discussion

Sepsis frequently arises from combat-related wounds and trauma, characterized as a perilous organ dysfunction stemming from an erratic host reaction to infection. Additionally, it ranks as a principal factor contributing to mortality rates and heightened healthcare expenditures within contemporary intensive care settings (23). Sepsis represents an abnormal systemic response to ordinary infections, typically characterized by a preliminary hyper-inflammatory phase followed by an immunosuppressive phase, leading to multiple organ dysfunction (5, 24). In the early stage of sepsis, cytokines like tumor necrosis factor (TNF), interleukin-1β (IL-1β), and interleukin-6 (IL-6) mediate the inflammatory reaction, initiating the systemic inflammatory response syndrome (SIRS) (25). In the later stages of sepsis, the immune response becomes suppressed, manifesting as compensatory anti-inflammatory response syndrome (CARS) (26). Biomarkers of this phase include anti-inflammatory cytokines and changes in the distinguishing markers on the surfaces of monocytes and lymphocytes. Integrating a range of pro-inflammatory and anti-inflammatory biomarkers may potentially facilitate the early detection of patients susceptible to developing severe sepsis, even before the occurrence of organ dysfunction. This approach could allow for prompt supportive intervention, enhancing patient outcomes, decreasing mortality rates, and reducing healthcare expenditures (5). Many existing biomarkers, such as C-reactive protein (CRP) and procalcitonin (PCT), although elevated during inflammatory responses, lack specificity and are influenced by other inflammatory or infectious conditions. Some biomarkers do not show significant changes during the progression of sepsis, rendering it challenging to capture the dynamic fluctuations and severity of the condition. Identifying and validating new biomarkers for sepsis can significantly enhance early diagnosis and treatment efficacy, thereby improving patient prognosis (27).

While the precise mechanisms are yet to be fully understood, immunosuppression is regarded as a significant contributor to mortality in sepsis (23). The mechanisms of sepsis-induced immunosuppression are highly complex, involving various cellular and molecular pathways. Immune checkpoint proteins, such as PD-1 and CTLA-4, are upregulated in sepsis, leading to immunosuppression by inhibiting T cell activation and proliferation (28–31). Regulating the expression and function of these checkpoint proteins is a current research focus. Autophagy fulfills a dual function within sepsis; it can protect cells by clearing damaged organelles and proteins, but excessive autophagy may lead to immune cell death, exacerbating immunosuppression (32). Ferroptosis, a type of cell death dependent on iron and characterized by lipid peroxidation, may further impair immune responses by disrupting the integrity of immune cell membranes during sepsis (33, 34). During sepsis, immune cells such as T cells, B cells, and natural killer cells undergo significant apoptosis, leading to a marked decline in immune system function and reduced resistance to infection (35–39). The count of regulatory T cells (Tregs) escalates, and these cells modulate immune responses through the secretion of anti-inflammatory cytokines like IL-10 and TGF-β (40–42). Sepsis also causes dysfunction in immune cells, including antigen-presenting cells like dendritic cells and macrophages, impairing antigen presentation and T cell activation (43, 44). Studies have shown that targeting immunosuppression can reverse immune cell dysfunction, providing a theoretical basis for developing new immunotherapeutic strategies (37, 45–48).

The identification of novel biomarkers has not only improved the diagnosis and treatment outcomes of sepsis but also provided new research directions for studying the mechanisms of sepsis-induced immunosuppression (27, 49). Studies have found that the levels of BMP9 are significantly reduced in sepsis patients, and these levels are closely related to patient prognosis. BMP9 can serve not only as a prognostic biomarker but also has potential value as a therapeutic target (50).

Traditional treatments for sepsis, such as fluid resuscitation and broad-spectrum antibiotics, although somewhat effective, have drawbacks including fluid overload, antibiotic resistance, and disruption of the gut microbiota (51–53). Personalized treatment, which adjusts therapeutic strategies based on the patient’s specific conditions through methods like gene expression analysis, single-cell transcriptomics, and dynamic monitoring, is gaining importance in sepsis management due to its potential to reduce side effects and improve outcomes (54–59). Machine learning models can predict the occurrence and progression of sepsis based on patients’ gene expression data (60). Researchers have developed machine learning classification models based on preoperative transcriptomic features to predict postoperative sepsis (61). These models can help clinicians more accurately assess patient risk and formulate personalized treatment plans.

In this study, we utilized machine learning and bioinformatics to identify new diagnostic biomarkers for sepsis and conducted a comprehensive analysis of immune cell infiltration characteristics, affording novel pathways for delving deeper into the intricacies of sepsis-induced immunosuppressive mechanisms. We merged two datasets (GSE69063 and GSE236713) from the Gene Expression Omnibus and removed batch effects. Between the control and the sepsis groups, our research revealed a total of 1443 DEGs, including 891 upregulated and 552 downregulated genes. According to the results of GO enrichment analysis, differentially expressed genes are mainly associated with T cell differentiation, immune response activation of cell surface receptors, innate immune response activation signal transduction, immune receptor activity, and extracellular matrix-related functions. The KEGG analysis is related to Th17 cell differentiation, Th1 and Th2 cell differentiation, T cell receptor signaling pathway, and Cytokine-cytokine receptor interaction. We extracted 262 genes commonly covered by WGCNA and DEGs, and employed three distinct machine learning algorithms to screen and identify diagnostic biomarkers associated with sepsis.

The Random Forest algorithm is an ensemble learning method that relies on decision trees (62). It combines multiple decision trees to form a forest, improving accuracy for classification or regression tasks (63). Using the Bagging algorithm (Bootstrap aggregating), it generates multiple subsets by sampling with replacement from the training set and trains individual decision trees. Finally, the overall prediction result is obtained by averaging or majority voting (64). The Random Forest algorithm is capable of generating highly precise classifiers across diverse datasets, managing extensive input variables efficiently, and supporting parallel processing (63). LASSO is a widely used regression analysis method in statistics. Its core idea is to compress coefficients to achieve variable selection and complexity adjustment, thereby improving the predictive accuracy and interpretability of the model (65, 66). SVM-RFE is a wrapper-based feature selection method. It starts with all potential genes (or other predictor variables) and then iteratively removes them one by one, forming a backward elimination process. During every cycle, genes are sorted in accordance with their significance in relation to the target variable, allowing identification of the most relevant genes and simplifying the model to improve predictive performance (67). Finally, we extracted 12 genes common to three machine learning algorithms: MS4A4A, AFF3, P2RY10, SIPA1L2, CD40LG, ST6GALNAC3, CD3E, LILRA5, KREMEN1, FCER2, CCNB2, and HJURP, which were identified again as potential biomarkers by the validation gene set. CIBERSORT is a widely used computational method in immunology research. It is based on a linear regression model that quantitatively analyzes the proportions of various immune cell subtypes in tissues using gene expression data (68). By training on known immune cell characteristic gene expression profiles, CIBERSORT estimates the relative abundance of immune cell subpopulations in mixed cell samples (69). The variation in immune cell infiltration, encompassing diverse types of immune cells, appears to correlate with the onset and progression of sepsis (37). To acquire profound insight into the role of immune cell infiltration in this context, we employed CiberSort for analysis.

Xuebijing injection is a traditional Chinese medicine injection that has shown significant efficacy in the management of sepsis over the past few years (70). Xuebijing works by modulating the body’s abnormal responses, protecting vascular endothelial cells, and mitigating the interaction between the inflammatory and coagulation systems, thereby preserving the physiological functions of major organs. Its application in the treatment of COVID-19 has also shown certain efficacy. Research indicates that Xuebijing can suppress the cytokine storm caused by the coronavirus, improve the Pneumonia Severity Index (PSI) of patients, and increase the cure rate (71). In a multicenter randomized double-blind placebo-controlled clinical trial, the 28-day all-cause mortality rate for the Xuebijing group was markedly reduced compared to the placebo group (18.8% versus 26.1%), highlighting its efficacy in potentially lowering sepsis-related mortality (72). Additionally, Xuebijing can improve patients’ immune function and reduce the incidence of multiple organ dysfunction syndrome (MODS). These findings provide high-level evidence for the application of Xuebijing in sepsis treatment. Astragalus, Salvia, and Safflower are the main components of Xuebijing (73), commonly used in the clinical treatment of sepsis. Using the Traditional Chinese Medicine Systems Pharmacology Database and Analysis Platform (TCMSP), we identified the chemical constituents of these three herbs and their target genes. We found that CD40LG is not only one of the 12 core genes we identified, but also a common target of the active components quercetin, luteolin, and apigenin in these herbs. We extracted the common chemical structure of these active ingredients—flavonoids. Through docking analysis, we further validated the interaction between flavonoids and CD40LG, providing strong evidence for personalized sepsis treatment. To further validate the practical application value of the gene in clinical settings and explore its roles in the pathogenesis of sepsis, we collected blood samples from healthy individuals and sepsis patients with or without the administration of Xuebijing for PBMC extraction, followed by qPCR and WB. At both gene and protein levels, CD40LG significantly decreased in sepsis patients without Xuebijing treatment compared to the healthy group, while CD40LG was partially rescued in sepsis patients with Xuebijing treatment.

CD40LG, predominantly located on the surface of T cells, serves as a critical co-stimulatory molecule in immune system functions. By attaching to the CD40 receptor on B cells and additional antigen-presenting cells, it facilitates B cell activation, antibody synthesis, and modulation of inflammatory processes (74, 75). This interaction is essential for B cell activation, antibody production, and the regulation of inflammatory pathways. CD40LG plays a multifaceted role in disease mechanisms, particularly in immune regulation and inflammation. It is a powerful modulator of inflammatory pathways, promoting the build-up of inflammatory white blood cells in atherosclerotic plaques and driving the expression of inflammatory genes. Produced by activated T lymphocytes and platelets, CD40LG can be converted into a soluble variant known as sCD40L, which behaves similarly to cytokines. Both the membrane-bound and soluble forms participate in inflammatory reactions and various immune and vascular disorders. Soluble CD40L, chiefly released by platelets, has been linked to harmful transfusion reactions, including transfusion-related acute lung injury (TRALI) (76). Genetic mutations in the CD40LG gene lead to X-linked hyper IgM syndrome (XHIM), a condition marked by the absence of T cell-dependent humoral immunity and specific IgG antibodies (77). A multicenter, prospective study showed that sCD40L levels might play a role in sepsis, with circulating sCD40L levels in septic patients significantly higher than those in the control group, and non-survivors having higher sCD40L levels than survivors (78). This is inconsistent with our bioinformatics analysis results, which may be due to differences in sample types and sources used in different studies. In the study by Pastor E et al., the measurement was of soluble CD40 ligand (sCD40L) levels in serum, whereas our bioinformatics analysis was based on CD40LG gene expression levels in whole blood samples. Nevertheless, the important role of CD40LG in sepsis cannot be denied. Further in-depth mechanistic exploration of changes in CD40L levels in serum and whole blood is still needed. The reduction of CD40LG may be associated with immunosuppression in late-stage sepsis patients (23). The interaction between CD40LG and CD40 receptors on B cells and other antigen-presenting cells is vital to the immune response. The decrease in CD40LG inhibits this immune response. After treatment with Xuebijing, the expression of CD40LG increases, which may be due to the binding of flavonoids to CD40LG, enhancing its stability, and possibly through signal transduction, increasing the gene transcription and protein expression of CD40LG.

The CD40LG may serve as a novel biomarker for the diagnosis of sepsis and as an indicator for evaluating treatment efficacy and provides a new research direction for further studying the mechanisms of immunosuppression in sepsis. However, our study has the following limitations. Machine learning models may overfit training data during training, yielding suboptimal outcomes when faced with fresh data sets. If the selected genes perform well only in the training data but poorly in other datasets, using these genes as diagnostic markers may be unreliable. Additionally, CIBERSORT estimates the composition and abundance of immune cells based on transcriptome data, but gene interactions within the organism and overlapping gene expression between different cell types may affect the accuracy of individual gene expression levels. To mitigate overfitting in machine learning models, we enhanced generalization through expanded sample size, data augmentation, feature selection (e.g., LASSO, random forest), regularization (L1, L2), cross-validation, and ensemble learning (e.g., XGBoost) (79). Future work will focus on robust algorithms for high-dimensional/small-sample data, multi-omics integration, and transparent model training. For CIBERSORT’s gene expression overlap issue, we plan to develop single-cell-based deconvolution tools and integrate multi-modal data (e.g., epigenetics, proteomics) to improve cell type resolution (80). Bias correction tools and multivariate regression will address confounding factors, ensuring result reliability (81). Therefore, the results of this study still need validation using larger datasets and extensive experiments to determine their reliability. Through biological experiments, we have confirmed that flavonoids can benefit sepsis patients by affecting CD40LG. However, the underlying mechanisms of this effect require further investigation.

In summary, our research has identified twelve genes, including CD3E, CD40LG, LILRA5, and FCER2, as potential diagnostic markers for sepsis. Among these, we selected CD40LG, a target gene for the three main components of Xuebijing, as one of the core genes. Through biological experiments, we concluded that Xuebijing treatment might improve the prognosis of sepsis patients by affecting CD40LG, providing a new research direction for further studying the mechanisms of immunosuppression in sepsis.

## Data Availability

The datasets supporting this study are available from GEO database (GSE69063, GSE236713, GSE137340, GSE28750 and GSE65682). The inquiries of corresponding code can be directed to the corresponding authors.

## Supplementary material

Supplementary Figure 1 Scatter plots showing gene expression data before and after batch correction.

Supplementary Figure 2
**(A)** Heatmap of the top 25 genes with the highest and lowest logFC among all statistically significant differentially expressed genes. **(B)** Volcano plot of the 1443 DEGs. **(C)** RF accuracy graph showing the relationship between the number of feature genes and diagnostic accuracy. RF: Random Forest; DEGs differentially expressed genes; FC fold-change.

Supplementary Figure 3 Scatterplot of GS for recurrence vs. MM in the other modules.

Supplementary Figure 4 Linkages between the other key genes and infiltrating immune cells.

Supplementary Figure 5 Validation in the GSE137340 Dataset **(A, B)** A boxplot depicts the expression levels of key genes between the pediatric sepsis group and the control group in the validation dataset; **(C-E)** The ROC curve for diagnostic efficacy validation in GSE137340 dataset. **(F)** A Venn diagram illustrates the overlapping genes with significant differences and AUC greater than 0.8 in GSE137340.

Supplementary Figure 6 Validation in the GSE28750 Dataset **(A-C)** A boxplot depicts the expression levels of key genes between the sepsis group and the control group in GSE28750 dataset; **(C-E)** The ROC curve for diagnostic efficacy validation in GSE28750.

Supplementary Figure 7 Quantitative PCR (qPCR) analysis of the mRNA expression of ST6GALNAC3, CD3E, LILRA5, KREMEN1, HJURP, CCNB2, FCER2, MS4A4A, AFF3, P2RY10 and SIPA1L2. Relative mRNA levels were measured in healthy control and sepsis using specific primers. (n = 6). Data are shown as mean ± SD. *P < 0.05, ****P < 0.0001 for indicated comparisons.

Supplementary Table 1 Information on microarray datasets obtained from GEO.

Supplementary Table 2 Primer information.
