# Supplementary figures and images for: Comprehensive integration of diagnostic biomarker analysis and immune cell infiltration features in sepsis via machine learning and bioinformatics techniques
Source: Front Immunol. 2025 Mar 10;16:1526174. doi: 10.3389/fimmu.2025.1526174 (PMC11931141; doi:10.3389/fimmu.2025.1526174)

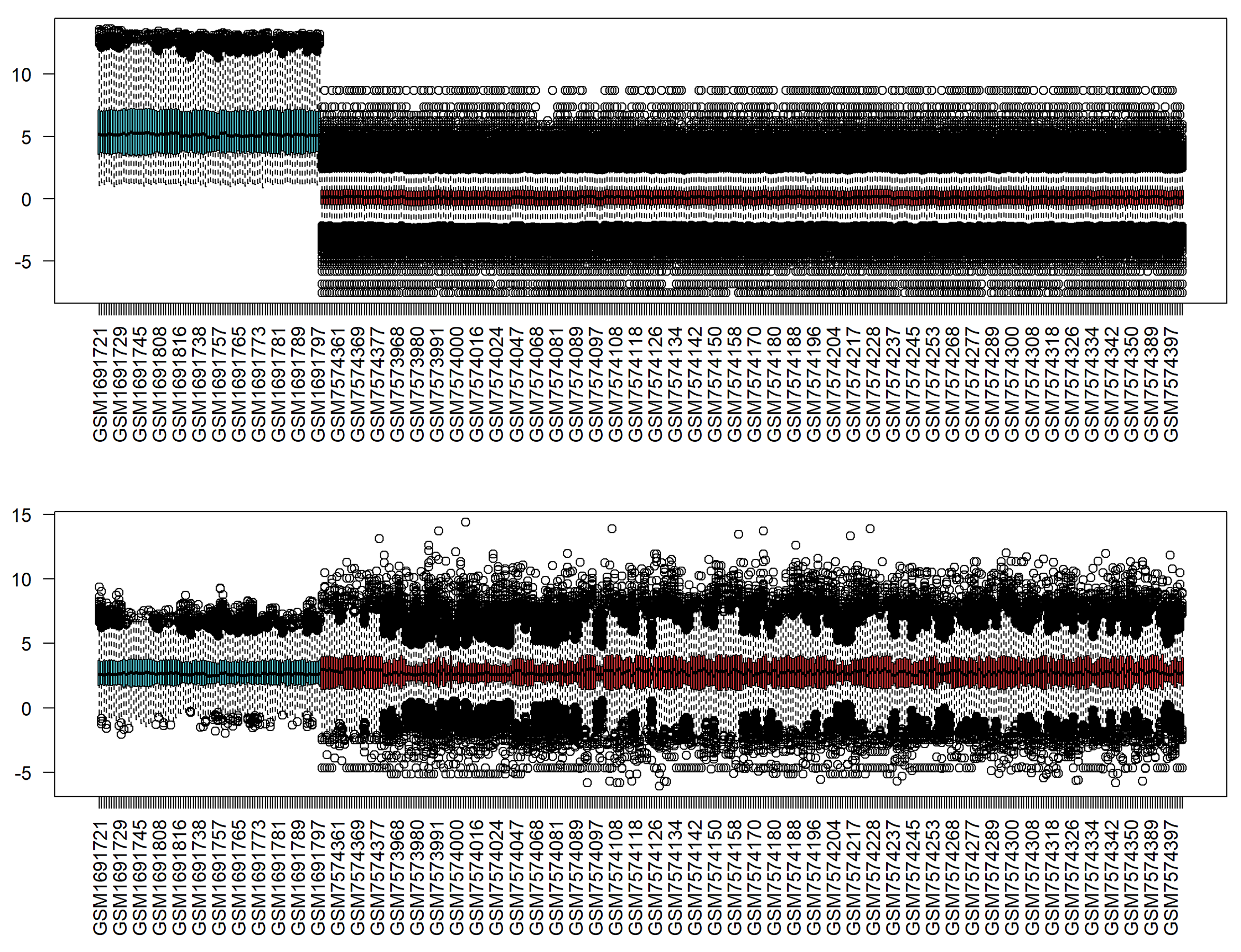

Supplement: Supplementary Figure 1 — Scatter plots showing gene expression data before and after batch correction. [file Image1.tif]

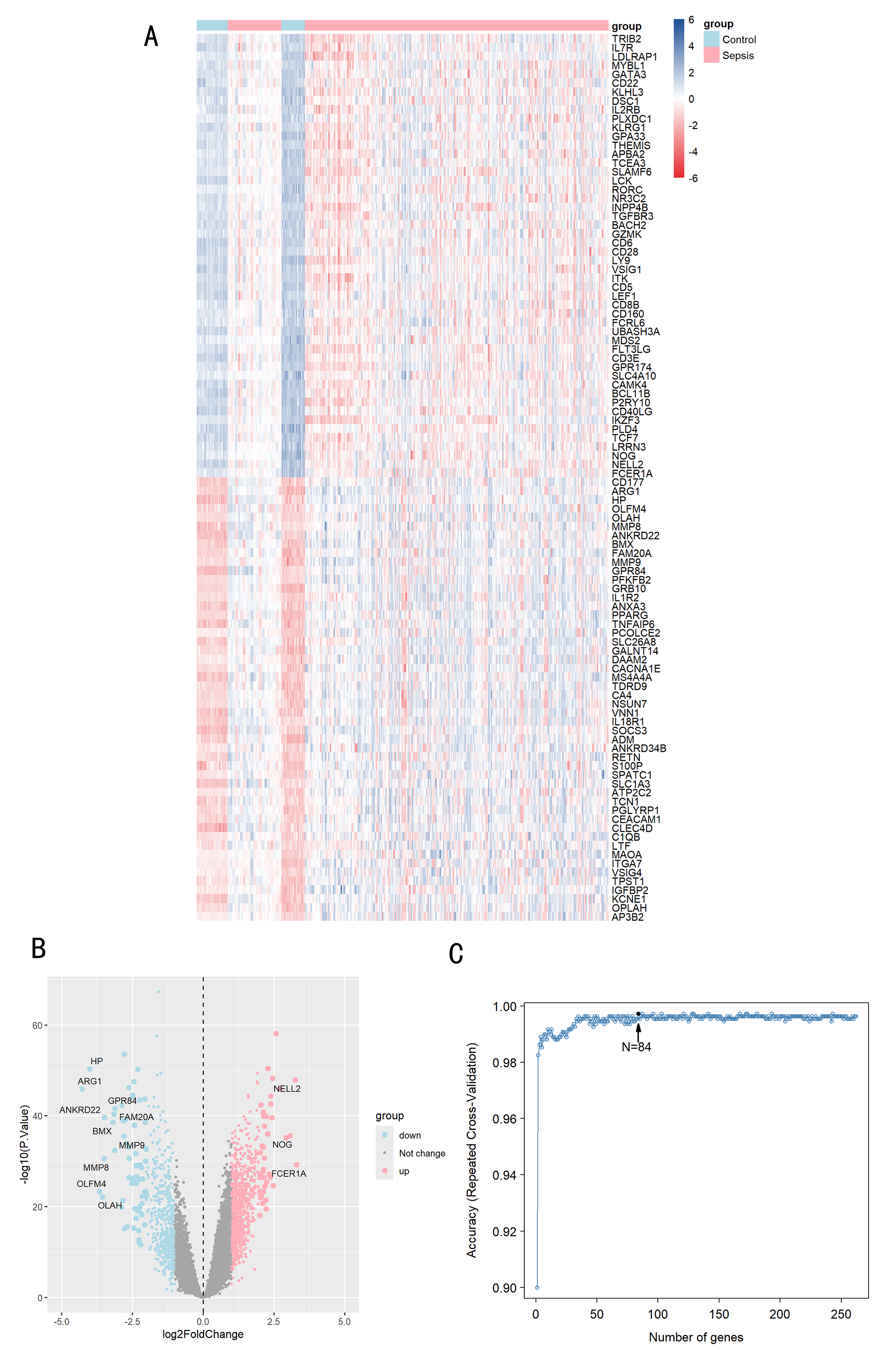

Supplement: Supplementary Figure 2 — (A) Heatmap of the top 25 genes with the highest and lowest logFC among all statistically significant differentially expressed genes. (B) Volcano plot of the 1443 DEGs. (C) RF accuracy graph showing the relationship between the number of feature genes and diagnostic accuracy. RF: Random Forest; DEGs differentially expressed genes; FC fold-change. [file Image2.tif]

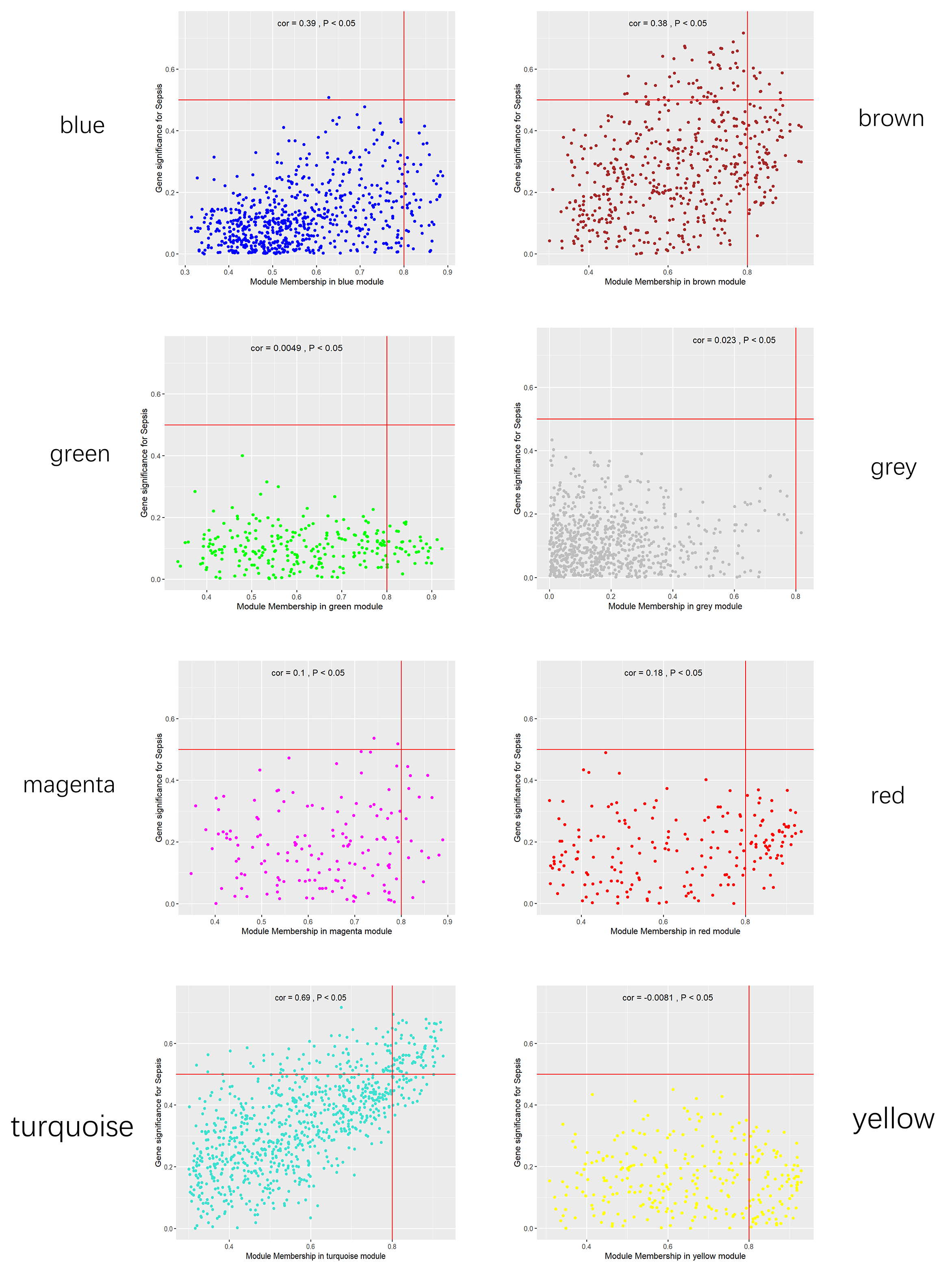

Supplement: Supplementary Figure 3 — Scatterplot of GS for recurrence vs. MM in the other modules. [file Image3.tif]

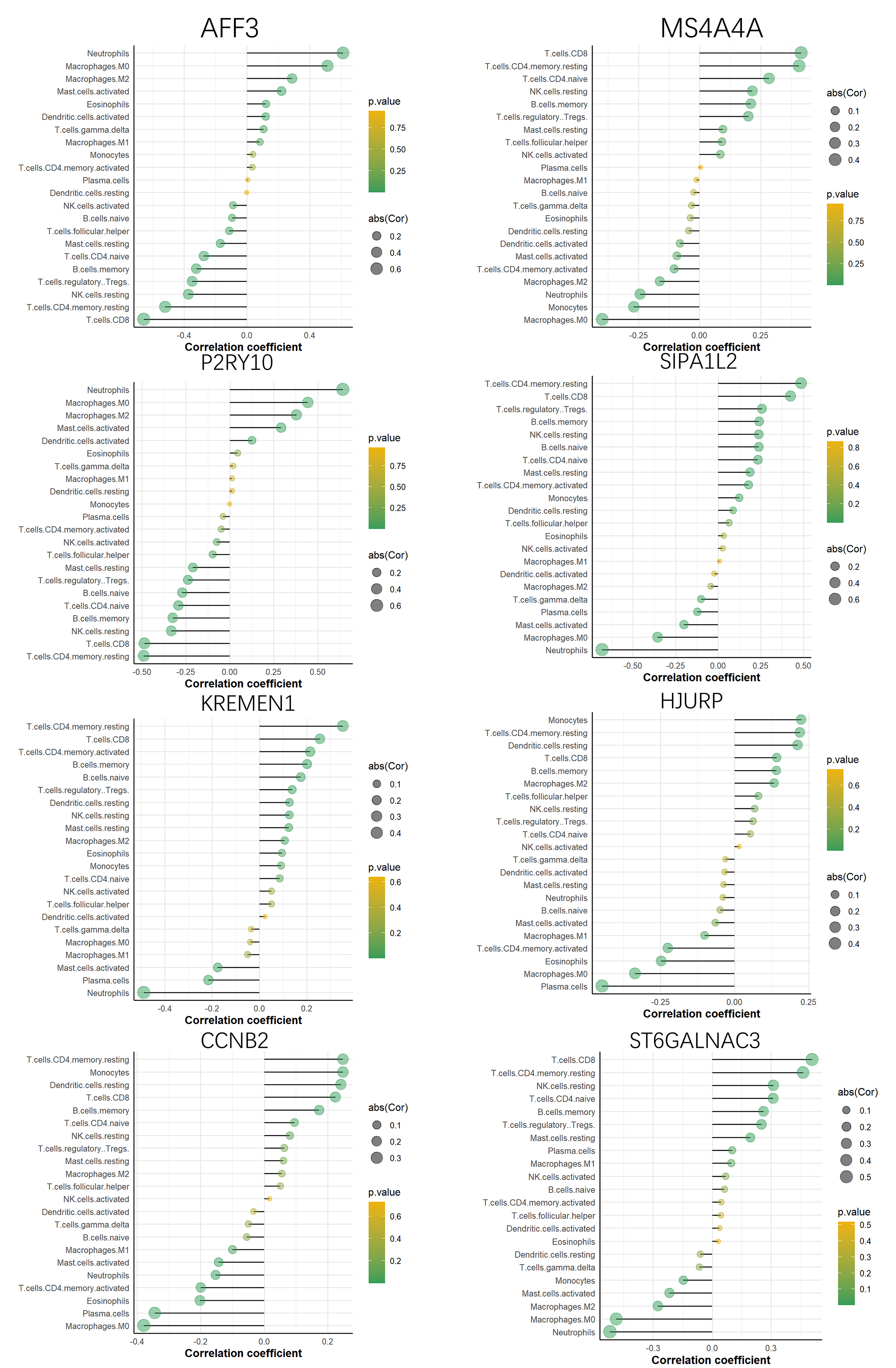

Supplement: Supplementary Figure 4 — Linkages between the other key genes and infiltrating immune cells. [file Image4.tif]

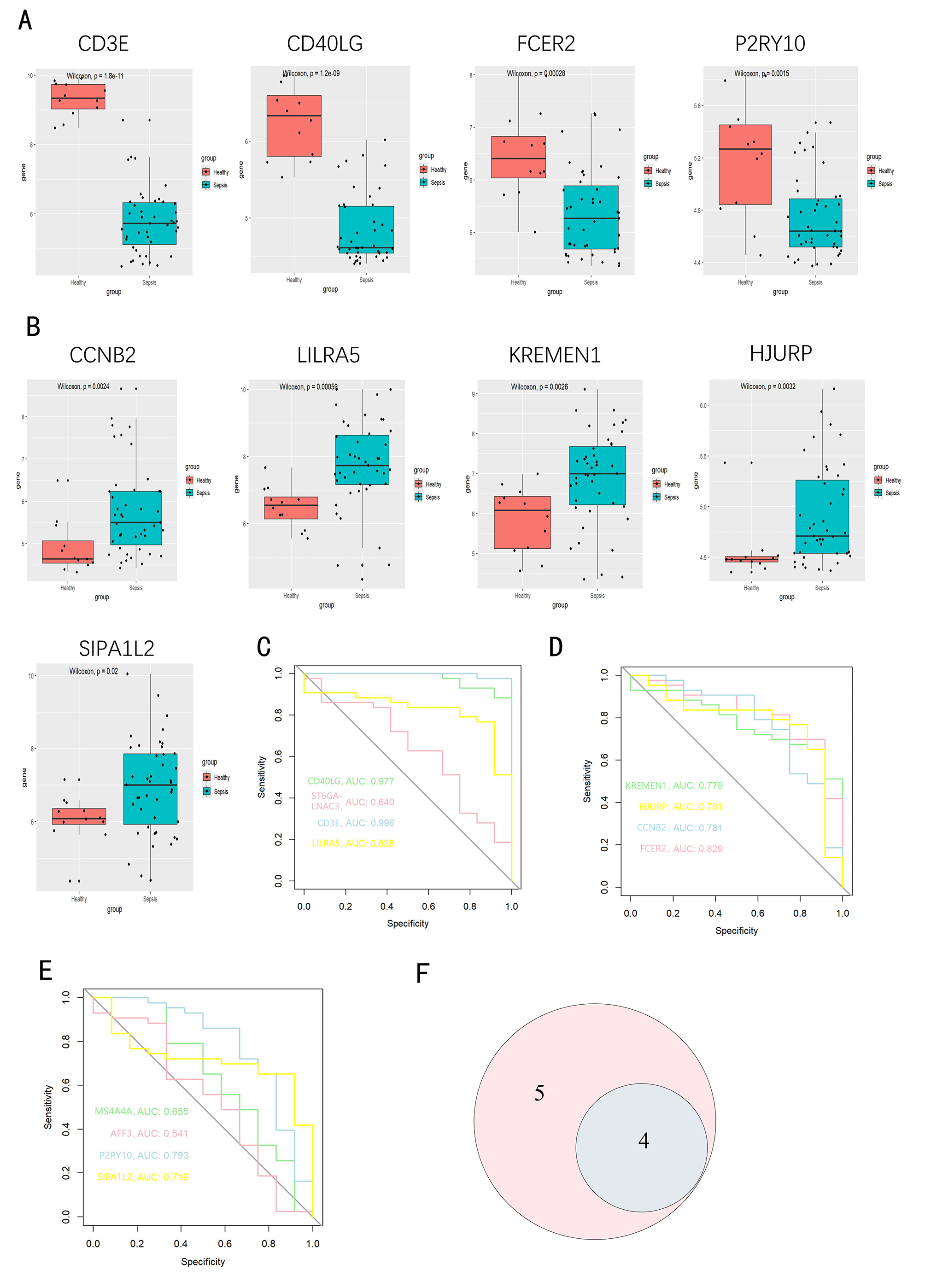

Supplement: Supplementary Figure 5 — Validation in the GSE137340 Dataset (A, B) A boxplot depicts the expression levels of key genes between the pediatric sepsis group and the control group in the validation dataset; (C-E) The ROC curve for diagnostic efficacy validation in GSE137340 dataset. (F) A Venn diagram illustrates the overlapping genes with significant differences and AUC greater than 0.8 in GSE137340. [file Image5.tif]

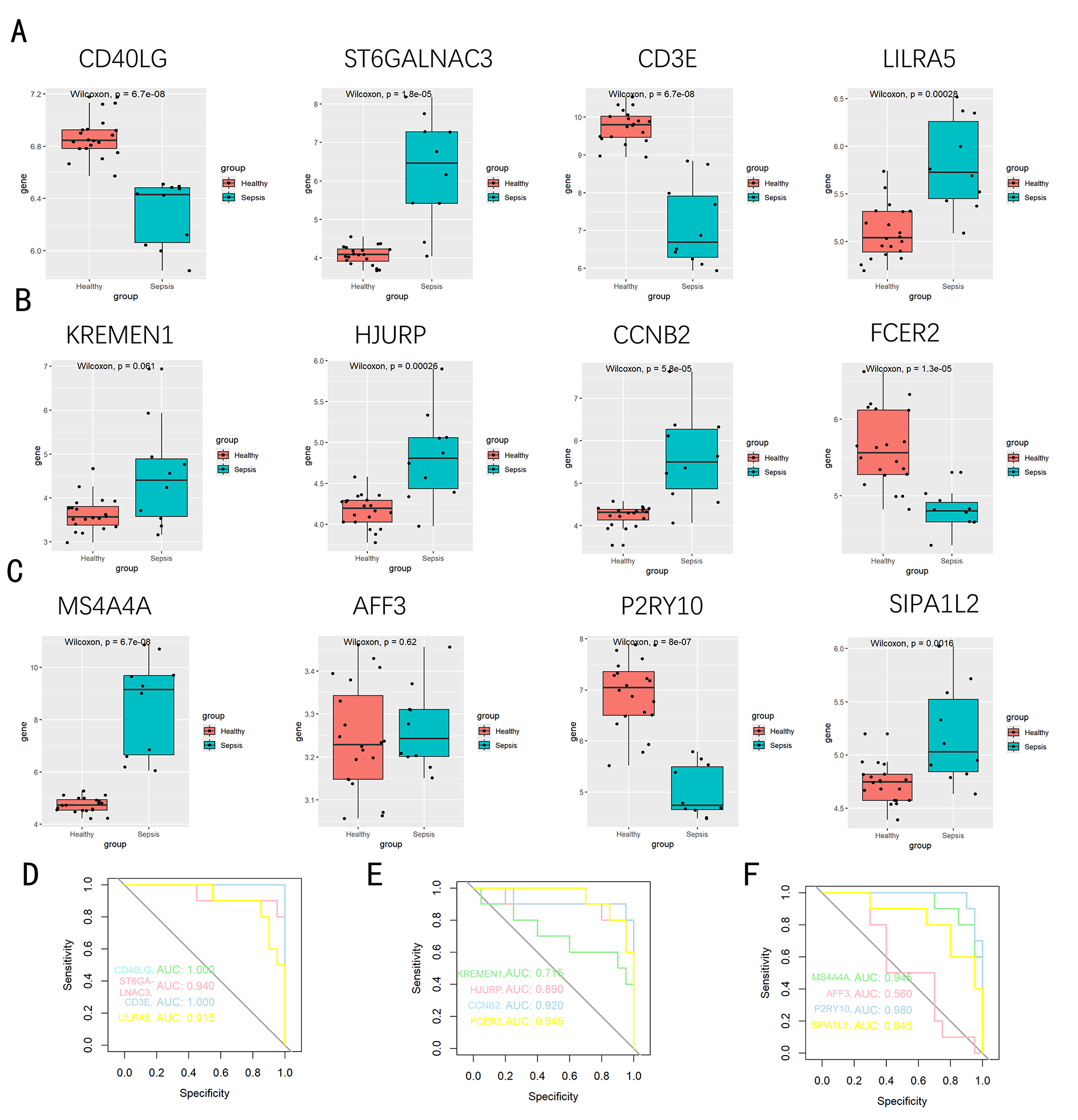

Supplement: Supplementary Figure 6 — Validation in the GSE28750 Dataset (A-C) A boxplot depicts the expression levels of key genes between the sepsis group and the control group in GSE28750 dataset; (C-E) The ROC curve for diagnostic efficacy validation in GSE28750. [file Image6.tif]

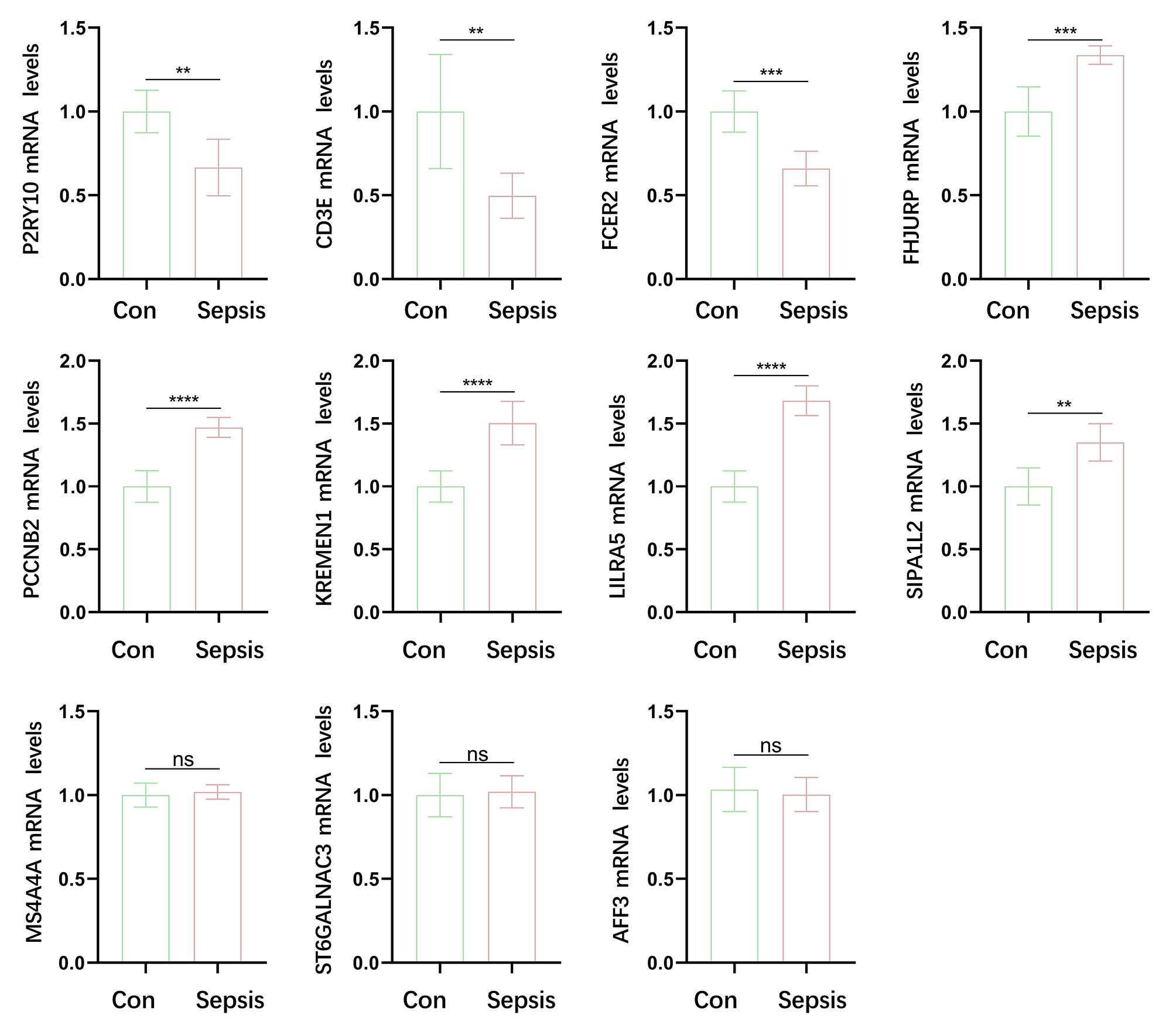

Supplement: Supplementary Figure 7 — Quantitative PCR (qPCR) analysis of the mRNA expression of ST6GALNAC3, CD3E, LILRA5, KREMEN1, HJURP, CCNB2, FCER2, MS4A4A, AFF3, P2RY10 and SIPA1L2. Relative mRNA levels were measured in healthy control and sepsis using specific primers. (n = 6). Data are shown as mean ± SD. *P < 0.05, ****P < 0.0001 for indicated comparisons. [file Image7.tif]

## Slide 1
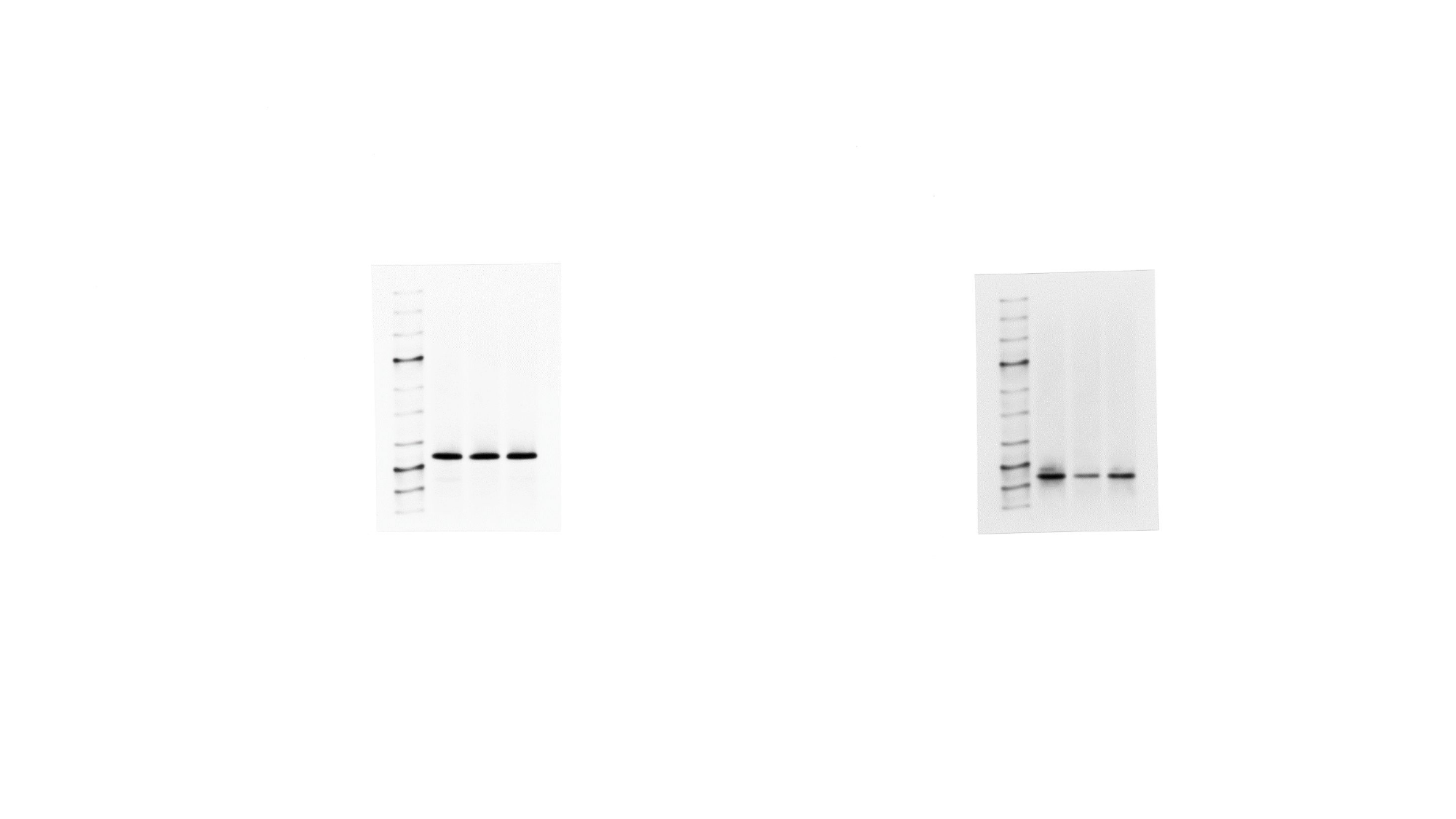

Supplement: Supplementary file 10 [file Presentation1.pptx]
