# Supplementary material for: Comprehensive integration of diagnostic biomarker analysis and immune cell infiltration features in sepsis via machine learning and bioinformatics techniques
Source: Front Immunol. 2025 Mar 10;16:1526174. doi: 10.3389/fimmu.2025.1526174 (PMC11931141; doi:10.3389/fimmu.2025.1526174)
Supplement: Supplementary Table 1 — Information on microarray datasets obtained from GEO. [file Table1.docx]

Supplementary table1 Information on microarray datasets obtained from GEO

| GEO dataset | Healthy control | sepsis |
| --- | --- | --- |
| GSE69063 | 33 | 38 |
| GSE236713 | 30 | 324 |
| GSE137340 | 27 | 12 |
| GSE28750 | 20 | 10 |
| GSE65682 | 42 | 760 |

Supplementary table2 Primer information

|  | F | R |
| --- | --- | --- |
| CD40LG | GCGGCACATGTCATAAGTGAGG | GTCCTTGTCTTTTAACGGTCAGC |
| ST6GALNAC3 | TACGTGACCACAGAGAAGCGCA | CGTGAATGCCATAACAGGCGTC |
| CD3E | CCAGGATACTGAGGGCATGT | CCAGGATACTGAGGGCATGT |
| LILRA5 | GCAGGTATGGTCAGAACCCAGT | GCGGATGAGATTCTCTACTGCG |
| KREMEN1 | CCTCCTCTAACTGGCACCAGTA | GCAAGCATAGCCTGACTCCATC |
| HJURP | CAGACCAGGAAGAGTCAGTTGC | CTTCCAGCTCTGTTACCTGCAC |
| CCNB2 | CAACCAGAGCAGCACAAGTAGC | GGAGCCAACTTTTCCATCTGTAC |
| FCER2 | GGTATGCCTGTGACGACATGGA | TTCAGGTCCAAGTTCCGAAGGC |
| MS4A4A | TGGATGGCATGGTGCTCCTCTT | GGAGATGCTGTTTCTGCCATGTG |
| AFF3 | CACCTGGCAAAGTGGAACCAAC | CGATGTGCCATTGTCTGGACTC |
| P2RY10 | CCTGGTCTTCTGGCTAACAGTG | TGAGCAAGGTCAGCCACAGAGA |
| SIPA1L2 | TGTCCTCGGTAGACAACTGTGC | TTCACATGGAAGCCAAGCTGGC |
